# *CACNA1I* gain-of-function mutations differentially affect channel gating and cause neurodevelopmental disorders

**DOI:** 10.1093/brain/awab101

**Published:** 2021-03-11

**Authors:** Yousra El Ghaleb, Pauline E Schneeberger, Monica L Fernández-Quintero, Stefanie M Geisler, Simone Pelizzari, Abeltje M Polstra, Johanna M van Hagen, Jonas Denecke, Marta Campiglio, Klaus R Liedl, Cathy A Stevens, Richard E Person, Stefan Rentas, Eric D Marsh, Laura K Conlin, Petronel Tuluc, Kerstin Kutsche, Bernhard E Flucher

**Affiliations:** 1 Institute of Physiology, Medical University Innsbruck, Innsbruck 6020, Austria; 2 Institute of Human Genetics, University Medical Center Hamburg-Eppendorf, Hamburg 20251, Germany; 3 Institute of Theoretical Chemistry, University of Innsbruck, Innsbruck 6020, Austria; 4 Department of Pharmacology, University of Innsbruck, Innsbruck 6020, Austria; 5 Department of Clinical Genetics, Amsterdam UMC, Vrije Universiteit Amsterdam, Amsterdam, 1012 WX, The Netherlands; 6 Department of Pediatrics, University Medical Center Hamburg-Eppendorf, Hamburg 20251, Germany; 7 Department of Pediatrics, University of Tennessee College of Medicine, Chattanooga, TN 37403, USA; 8 GeneDX, Gaithersburg, MD 20877, USA; 9 Division of Genomic Diagnostics, Children’s Hospital of Philadelphia, Philadelphia, PA 19104, USA; 10 Division of Neurology, Children’s Hospital of Philadelphia, Philadelphia, PA 19104, USA; 11 Department of Pathology and Laboratory Medicine, Perelman School of Medicine, University of Pennsylvania, Philadelphia, PA 19104, USA

**Keywords:** low-voltage-gated calcium channels, T-type calcium channels, Ca_V_3.3, epilepsy, intellectual disability (ID)

## Abstract

T-type calcium channels (Ca_v_3.1 to Ca_v_3.3) regulate low-threshold calcium spikes, burst firing and rhythmic oscillations of neurons and are involved in sensory processing, sleep, and hormone and neurotransmitter release. Here, we examined four heterozygous missense variants in *CACNA1I*, encoding the Ca_v_3.3 channel, in patients with variable neurodevelopmental phenotypes. The p.(Ile860Met) variant, affecting a residue in the putative channel gate at the cytoplasmic end of the IIS6 segment, was identified in three family members with variable cognitive impairment. The *de novo* p.(Ile860Asn) variant, changing the same amino acid residue, was detected in a patient with severe developmental delay and seizures. In two additional individuals with global developmental delay, hypotonia, and epilepsy, the variants p.(Ile1306Thr) and p.(Met1425Ile), substituting residues at the cytoplasmic ends of IIIS5 and IIIS6, respectively, were found. Because structure modelling indicated that the amino acid substitutions differentially affect the mobility of the channel gate, we analysed possible effects on Ca_v_3.3 channel function using patch-clamp analysis in HEK293T cells. The mutations resulted in slowed kinetics of current activation, inactivation, and deactivation, and in hyperpolarizing shifts of the voltage-dependence of activation and inactivation, with Ca_v_3.3-I860N showing the strongest and Ca_v_3.3-I860M the weakest effect. Structure modelling suggests that by introducing stabilizing hydrogen bonds the mutations slow the kinetics of the channel gate and cause the gain-of-function effect in Ca_v_3.3 channels. The gating defects left-shifted and increased the window currents, resulting in increased calcium influx during repetitive action potentials and even at resting membrane potentials. Thus, calcium toxicity in neurons expressing the Ca_v_3.3 variants is one likely cause of the neurodevelopmental phenotype. Computer modelling of thalamic reticular nuclei neurons indicated that the altered gating properties of the Ca_v_3.3 disease variants lower the threshold and increase the duration and frequency of action potential firing. Expressing the Ca_v_3.3-I860N/M mutants in mouse chromaffin cells shifted the mode of firing from low-threshold spikes and rebound burst firing with wild-type Ca_v_3.3 to slow oscillations with Ca_v_3.3-I860N and an intermediate firing mode with Ca_v_3.3-I860M, respectively. Such neuronal hyper-excitability could explain seizures in the patient with the p.(Ile860Asn) mutation. Thus, our study implicates *CACNA1I* gain-of-function mutations in neurodevelopmental disorders, with a phenotypic spectrum ranging from borderline intellectual functioning to a severe neurodevelopmental disorder with epilepsy.

## Teaser

T-type calcium channels regulate neuronal firing, are implicated in brain disease and are targets for anti-epileptic drugs. El Ghaleb *et al.* identify heterozygous missense variants of *CACNA1I* associated with variable neurodevelopmental disorders and epilepsy, and show how gain-of-function mutations cause hyper-excitability.

## Introduction

Low voltage-activated, T-type calcium channels (Ca_v_3) are expressed throughout the vertebrate nervous system and critical for normal cerebellar, thalamic, and cortical functions.^1-3^ Owing to their specific biophysical properties they regulate neuronal excitability and contribute to neural processing of pain, sensory, and motor functions, to neurotransmitter and hormone release, and to sleep.^4^ In addition, T-type calcium channels are also expressed in astrocytes.^5^ Mutations in *CACNA1G*, *CACNA1H*, and *CACNA1I* encoding Ca_v_3 channels have been associated with a range of neurodevelopmental, neurological, and/or psychiatric disorders.^6^ Accordingly, T-type calcium channels are regarded as promising candidate targets in ongoing drug development ventures.^7^

T-type calcium channels operate at negative voltages near the resting potential of nerve cells, where they regulate excitability and the rhythmic activity of neuronal circuits.^4^^,^^8^^,^^9^ At rest, the great majority of T-type channels are inactivated. Following hyperpolarization, they recover and generate low threshold calcium spikes and rebound burst firing. Because of their slow deactivation kinetics considerable amounts of calcium can enter nerve cells in the wake of an action potential. Furthermore, because of the negative voltage-dependence of activation and the incomplete overlap with the voltage-dependence of inactivation, a small fraction of T-type channels remains open at rest.^10^ In electrophysiological analyses these currents upon repolarization and at rest are evident as transient ‘tail currents’ and as continuous ‘window currents’, respectively. When aberrantly enlarged, both these currents can become the source of an increased calcium load threatening normal development and survival of neurons.

In recent years several disease-associated Ca_v_3 channel variants have been identified and functionally characterized in heterologous expression systems and genetic mouse models.^6^ The most deleterious variants detected in the *CACNA1G* and *CACNA1H* genes, encoding Ca_v_3.1 and Ca_v_3.2, respectively, represent *de novo* gain-of-function missense mutations causing congenital severe motor and cognitive impairment with cerebellar atrophy and primary aldosteronism, respectively.^11–13^ These missense mutations are primarily located at the cytoplasmic end of the channels_**’**_ S6 helices, which comprise the channel gate.^6^^,^^14^ Accordingly, the common feature of these mutations is that they affect the channel gating properties; foremost by slowing the kinetics and left-shifting the voltage-dependence of activation and inactivation. This leads to prolonged channel openings and increased window currents, which in turn result in hyper-excitability of neurons and an increased calcium load, which might be causal for neurological defects on one hand and aldosterone production and hypertension on the other.^6^ Involvement of both the *CACNA1G* and *CACNA1H* genes in inherited epilepsy has been proposed based on various findings in humans and mice.^15–17^ However, to date, none of the investigated variants have been undoubtable identified as causing seizure phenotypes and are rather classified as genetic risk factors for developing epilepsy (reviewed in Lory^6^).

The third member of the T-type channel family, Ca_v_3.3 encoded by *CACNA1I*, was identified as genetic risk factor in schizophrenia.^18–23^ The schizophrenia-associated missense variant p.(Arg1346His) causes decreased membrane expression and current density when expressed in heterologous cells, while other current properties remained unaltered.^24^ Computer simulation suggests that such reduced current density eliminates rebound burst firing in thalamic reticular nucleus (TRN) neurons, in which Ca_v_3.3 channels are highly expressed.^25^^,^^26^ Knock-in mice homozygous for the orthologous p.(Arg1346His) variant showed altered excitability of TRN neurons and deficits in sleep spindle occurrence.^27^ Thus, *CACNA1I* loss-of-function variants disrupt neuronal excitability and network activity and may contribute to the development of schizophrenia, autism and/or other complex neuropsychiatric disorders.^22^^,^^28^^,^^29^

Here we report three unrelated patients and one family with three affected individuals with heterozygous missense variants in the *CACNA1I* gene and variable neurodevelopmental phenotype. While the mother and her two children of one family showed variable cognitive impairment, the three unrelated probands had a severe phenotype with global developmental delay, hypotonia and epilepsy. Three of the four disease-associated missense variants affect an amino acid residue located at the cytoplasmic end of an S6 helix constituting the gate of the Ca_v_3.3 calcium channel. The fourth amino acid change is located in the closely adjacent region of an S5 helix. Structure modelling suggests that the amino acid substitutions reduce the mobility of the gate by introducing new stabilizing hydrogen bond interactions. Electrophysiological analysis demonstrated slowed kinetics and left-shifted voltage-dependence of activation and inactivation of all the Ca_v_3.3 mutant channels. These altered gating properties result in hyper-excitability, prolonged calcium currents, and a shift of firing modes when tested in the TRN neuron model and upon heterologous expression in mouse chromaffin cells. Interestingly, Ca_v_3.3 mutant channels with p.(Ile860Asn) or p.(Ile860Met) greatly differ in the magnitude of their effects on gating properties and cellular excitability, and these differences parallel the disease severity in the affected individuals. Thus, the data presented here provide evidence for a causal link of pathogenic missense variants in the *CACNA1I* gene with a range of neurodevelopmental phenotypes. The gain-of-function effects caused by the amino acid substitutions offer possible functional and mechanistic explanations for the pathophysiological role of the Ca_v_3.3 T-type calcium mutant channels in causing impaired cognitive function and epilepsy.

## Materials and methods

### Patients

Informed consent for genetic analyses was obtained for all patients, and genetic studies were performed clinically or as approved by the Institutional Review Boards of the relevant institutions. The patients or patients’ parents provided written informed consent for the participation in the study, clinical data and specimen collection, genetic analysis, and publication of relevant findings.

### Whole-exome sequencing

Quad whole-exome sequencing (WES) was performed in Family 2 (Patients 2–4 and the father of the siblings), trio WES in Families 1 and 3 (Patients 1, 5 and their parents) and duo WES in Family 4 (Patient 6 and mother). Variant validation was performed by Sanger sequencing with DNA obtained from leucocytes of patients and parents.

### Structure modelling

We predicted the structure of the wild-type T-type channel Ca_v_3.3 α_1_-subunit and the mutants in the activated state by building a homology model based on the cryo-electron microscopy (EM) structure of the Ca_v_3.1 α_1_-subunit in the inactivated state characterized by depolarized voltage-sensing domains and a closed intracellular gate (PDB accession code: 6KZO).^14^ The high sequence similarity of ∼85% allows a reliable structure prediction of the Ca_v_3.3 α_1_-subunit. Additionally, we also generated a homology model of the Ca_v_3.3 α_1_-subunit in a resting state based on the cryo-EM resting state structure of Na_v_Ab (PDB accession code: 6P6W).^30^

### Expression plasmids and transfections

The human Ca_v_3.3 subunit (Genebank ID AF393329)^31^ was transferred into an expression plasmid with an N-terminal GFP tag and the mutations were introduced by splicing by overlap extension (SOE)-PCR. HEK293T cells were transfected with the expression plasmids using FuGENE^®^ HD reagent (Promega). Chromaffin cells from 6–8-week-old male mice were obtained as described previously^32^^,^^33^ and transfected by electroporation with the Mouse Neuron Nucleofector^TM^ Kit.^34^

### Electrophysiology and data analysis

Calcium currents in HEK293T cells were recorded with the whole-cell patch-clamp technique in voltage-clamp mode. Patch pipettes had resistances between 1.8 MΩ and 4.5 MΩ when filled with (in mM) 135 CsCl, 1 MgCl_2_, 10 HEPES, 4 ATP-Na_2_ and 10 EGTA (pH 7.4 with CsOH). The extracellular bath solution contained (in mM) 2 CaCl_2_, 165 choline-chloride, 10 HEPES, and 1 MgCl_2_ (pH 7.4 with CsOH). All five experimental groups were analysed in transiently transfected cells from four to six independent cell passages. The variants were always recorded in parallel with the wild-type Ca_v_3.3 in cells of the same passage to obtain matched controls for statistical comparison. The means, standard error of the mean (SEM), and *P*-values of the experiments where the variants have separate control groups were calculated using the Student’s *t*-test, two-tailed, with significance criteria *P < 0.05, **P < 0.01, ***P < 0.001 and ****P < 0.0001. *P*-values of the experiments where the variants are compared to the same control group were calculated using the ANOVA and Tukey’s or Dunnett’s *post hoc* test.

The whole cell current-clamp recordings of isolated mouse chromaffin cells were performed in perforated-patch mode. Patch pipettes had a resistance between 1.8 MΩ and 4 MΩ when filled with (in mM): 10 NaCl, 10 KCl, 76 K_2_SO_4_, 1 MgCl_2_, 5 HEPES (pH 7.35 with KOH) and supplemented with 240 µg/ml amphotericin B. The external bath solution contained (in mM): 140 NaCl, 3.6 KCl, 2 NaHCO_3_, 0.5 NaH_2_PO_4_, 0.5 MgSO_4_, 2.5 CaCl_2_, 5 HEPES, 5 glucose (pH 7.4 with NaOH). All three experimental groups were analysed in eGFP-positive chromaffin cells from three independent culture preparations. Wild-type Ca_v_3.3, I860N and I860M were recorded in parallel on the same days to match controls and mutants for optimal statistical comparison. Data-points in scatter plots represent values of individual cells and means (line) ± standard error (SE). *P*-values were calculated using the Student’s *t*-test or ANOVA with Holm-Sidak *post hoc* test with significance criteria *P < 0.05, **P < 0.01, and ***P < 0.001.

### Computer model

Modelling was performed in the NEURON simulation environment^35^ using the model for thalamic relay neurons^36^ from the model database at Yale University (https://senselab.med.yale.edu/modeldb/ accessed 21 May 2021). The electrophysiological properties of the Ca_v_3.3 channels were modelled using Hodgkin-Huxley equations as described previously.^36^^,^^37^ The values of native T-type channels were substituted by the experimentally obtained values for the wild-type and the individual mutants.

Further details of the experimental procedures can be found in the Supplementary material.

### Data availability

The authors declare that all data supporting the findings of this study are available within the paper and its Supplementary material. Consent restrictions preclude sharing of full datasets, and the consents do not cover the deposition of the next-generation sequencing data in a public database.

## Results

### Heterozygous missense variants in the *CACNA1I* gene cause a spectrum of neurological symptoms

Patient 1 is an 8-year-old female, who is the only child of healthy unrelated parents (Supplementary Table 1). Pregnancy was complicated by reduced foetal movements, breech presentation, and mild gestosis. She was born at 37 + 6 weeks of gestation by caesarean section; her birth parameters were in the normal range. Muscular hypotonia was noted at age 4 weeks, and seizures were discussed because of atypical head movements. At the age of 4 months, she received anticonvulsive therapy because of pathological EEG findings associated with possible epileptic apnoea. She showed non-epileptic hyperexcitability, and within the second year of life epileptic myoclonus was noted. Between the age of 4 and 6 years, startle seizures triggered by noise were dominating with myoclonic seizures followed by a tonic episode. Last examination at age 8 years and 1 month revealed severe global developmental delay and severe proximal muscular hypotonia and distal muscular hypertonia; she did not show any interaction. She did not achieve any motor milestones and had no speech and no cognitive development. She had myoclonic seizures daily as the predominant seizure type and rare grand mal seizures that did not respond to treatment. Cortical blindness was diagnosed. She had severe obstructive sleep apnoea syndrome that required intermittent non-invasive ventilation at the age of 6 years. She received a gastrostomy tube because of feeding difficulties. Brain MRI at age 4 months was normal. At the age of 2 years and 7 months, brain imaging revealed frontal brain atrophy, a flattened brainstem and mildly delayed myelination. We performed trio WES in Patient 1 and her parents, and did not detect any biallelic variant that could underlie her severe neurodevelopmental phenotype. However, we identified the Sanger-validated *de novo* variant c.2579T>A in the *CACNA1I* gene predicting the amino acid substitution p.(Ile860Asn) as the only potentially disease-causing variant (Supplementary Tables 1 and 2).

Through GeneMatcher,^38^ we identified five additional individuals with a heterozygous missense variant in *CACNA1I*: Family 2 with three affected members (Patients 2–4), and the two unrelated Patients 5 and 6. We included these cases in the study for clinical comparison and functional analysis. Family 2 comprised a 58-year-old female (Patient 2), her 28-year-old son (Patient 3) and 31-year-old daughter (Patient 4) who all carried the *CACNA1I* variant c.2580C>G/p.(Ile860Met) (Supplementary Tables 1 and 2). The mother of Patients 3 and 4 had mild cognitive impairment, and she developed seizures at the age of 58 years. Patient 3 had mild intellectual disability, and Patient 4 showed moderate intellectual disability and speech retardation. Brain CT in Patient 2 did not reveal any abnormalities (Supplementary Table 1). Interestingly, in Patient 1 and Family 2 codon 860 of *CACNA1I* was affected resulting in substitution of isoleucine for asparagine in Patient 1 and for methionine in Family 2 (Supplementary Table 2). WES in female Patient 5 and male Patient 6 revealed the heterozygous *CACNA1I* variant c.3917T>C/p.(Ile1306Thr) and c.4275G>A/p.(Met1425Ile), respectively (Supplementary Tables 1 and 2). *De novo* occurrence of the variant was confirmed in Patient 5, while the change was absent in the mother of Patient 6, but his father was not available for testing (Supplementary Table 1). The 21-month-old Patient 5 and the 8-year-old Patient 6 presented with severe developmental delay, hypotonia, severe speech impairment, and cortical visual impairment. Seizures began at age 2 weeks in Patient 5 and at 2 years in Patient 6 and are controlled on medication. Brain imaging at the age of 12 months was unremarkable in Patient 5; in Patient 6, several brain abnormalities were observed at the age of 5 years (Supplementary Table 1). Similar to Patient 1, Patients 5 and 6 had feeding issues with reflux that required G-tube placement in Patient 5. Patients 1 and 6 presented with postnatal growth retardation, and Patient 6 in addition had growth hormone deficiency (Supplementary Table 1).

*CACNA1I* is intolerant to functional genetic variation (*Z*-score: 5.05; observed/expected value for missense variants: 0.59),^39^^,^^40^ and the four different *CACNA1I* missense variants affect three evolutionary conserved amino acid residues that were predicted to be intolerant to variation (Supplementary Fig. 1 and Table 2).^41^ All variants are absent from gnomAD, and three *in silico* algorithms predicted the missense variants to have a damaging impact on protein function (Supplementary Table 2). In summary, based on the absence of the identified *CACNA1I* variants in the population databases, a predicted deleterious effect of the missense variants on protein function, and impaired cognitive function in all six affected individuals, we believed the heterozygous *CACNA1I* missense changes to underlie the phenotype in all of them.

### Homology structure modelling predicts increased stability of activation gates in Ca_v_3.3-I860N, -I860M and -I1306T

**Figure 1:**
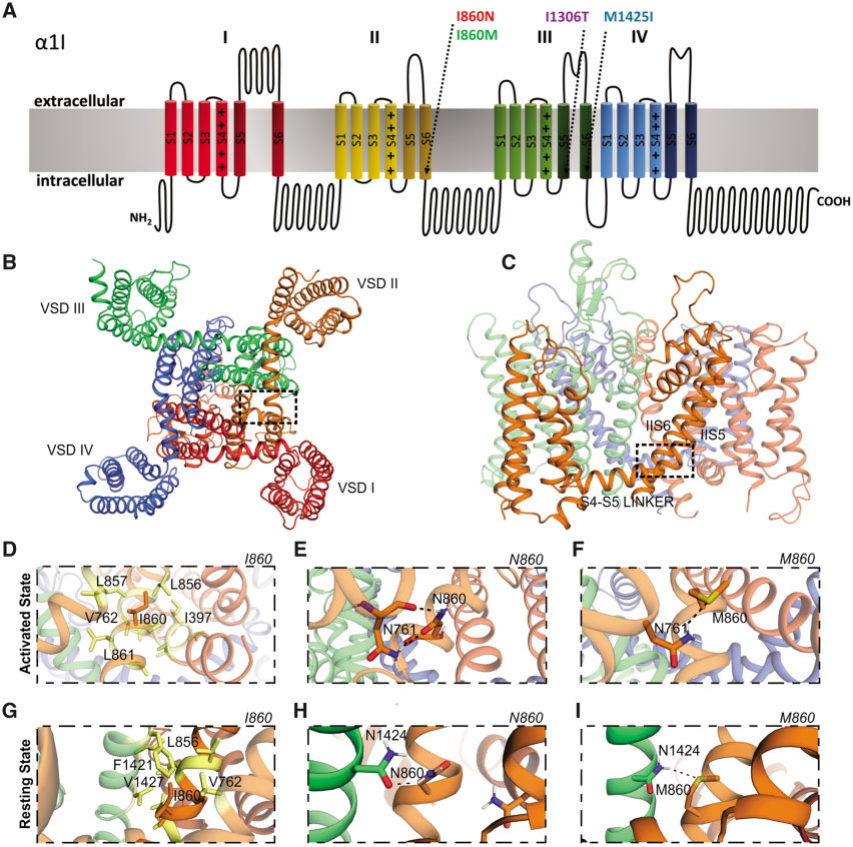
**Structure modelling of the variants I860N and I860M in Ca_v_3.3.** (**A**) Domain structure of Ca_v_3.3 indicating the location of the four pathogenic missense variants at the cytoplasmic end of transmembrane helices IIS6, IIIS5, and IIIS6 (arrows) which form part of the channel’s activation gate. (**B**–**F**) Structure homology model of Ca_v_3.3 based on the cryo-EM structure of Ca_v_3.1 in the activated state, colour-coded as in **A**. (**B**) Bottom view and (**C**) side view, frames show the areas depicted in **D**–**I**. VSDI–IV = voltage-sensing domains. (**D**) In wild-type, I860 in IIS6 forms numerous hydrophobic interactions with neighbouring residues in the IIS5, IIS6 and the S4-S5 linker. (**E**) The N860 variant forms two strong, stabilizing hydrogen bonds between N860 and N761 in the S4-S5 linker. (**F**) The M860 variant forms one weak sulphur hydrogen bond with N761. (**G**–**I**) Interactions of the different variants in the resting state. (**G**) Wild-type I860 forms numerous hydrophobic interactions with neighbouring residues in IIIS6 and IIS5. (**H**) The N860 variant forms one strong stabilizing hydrogen bond between N860 and N1424 in IIIS6. (**I**) The M860 variant forms one sulphur hydrogen bond with N1424.

To assess the structural impact of the missense variants, we generated homology structure models of wild-type and mutant Ca_v_3.3. Eukaryotic Ca_v_ channels are composed of four homologous repeats (I–IV), each comprising six membrane-spanning helices (S1–S6) (Fig. 1A). Helices S1–S4 of each repeat form separate voltage-sensing domains, while the four S5 and S6 helices and the connecting pore-loops form the channel pore with the selectivity filter and the activation gate (Fig. 1B and C). Because the two different substitutions of I860 (p.I860N and p.I860M) were found in individuals with greatly differing disease severity, we first focused our attention on these two variants. The I860 residue is located at the intracellular end of the IIS6 helix, a position known to be part of the channel activation gate (Fig. 1A–C).^42^ The structure homology model of the activated state of Ca_v_3.3, based on the cryo-EM structure of Ca_v_3.1 in the activated state,^14^ shows that I860 in wild-type Ca_v_3.3 forms multiple hydrophobic interactions with neighbouring residues in the IIS5, IIS6 and the S4–S5 linker (Fig. 1D). These hydrophobic interactions in the activation gate area have only a weak stabilizing effect, which allows the flexibility necessary for opening and closing the channel gate. When the I860 residue is replaced by asparagine (N860), the model predicts the formation of two strong hydrogen bonds between N860 and N761 in the S4–S5 linker (Fig. 1E). This stabilizes the activation gate in the activated state and probably perturbs S6 and S4–S5 linker movement upon deactivation and inactivation. Replacing the I860 residue with methionine (M860) results in the formation of a single stabilizing sulphur hydrogen bond between M860 and N761 (Fig. 1F). This sulphur hydrogen bond is considerably weaker than either one of the classical hydrogen bonds formed by the I860N variant. Thus, M860 may also stabilize the activation gate in the activated state and potentially perturb S6 and S4–S5 linker movement, but to a lesser extent than the I860N variant (Table 1).

**Table 1:** **Predicted interaction partners**‘ **of all four *CACNA1I* variants (N860, M860, T1306, I1425) and their corresponding wild-type residues (I860, I1306, M1425) in the activated and resting state**

**Variant**	**Interaction partners activated state**	**Interaction partners resting state**	**Nature of interaction**	**Stabilization**
I860	I397, V762, L856, L857, L861	V762, L856, F1421, V1427	Hydrophobic	Low
N860	N761	N1424	2 H-bonds	High
M860	N761	N1424	1 S-H bond	Medium
I1306	V1429	V1726	Hydrophobic	Low
T1306	E1432	N1723	Charged H-bond	High
M1425	V1310, I1309	I1306	Hydrophobic	Low
I1425	V1310, I1309	I1306	Hydrophobic	Low

Next we analysed possible interactions of wild-type I860 and mutant N860 and M860 in a homology model of Ca_v_3.3 in the resting state, based on the resting state structure of the prokaryotic sodium channel Na_v_Ab^30^ (Fig. 1G–I). Again, wild-type I860 formed multiple hydrophobic interactions (Fig. 1G). In contrast, N860 (Fig. 1H) and M860 (Fig. 1I) formed a single hydrogen bond and a sulphur hydrogen bond, respectively, with an asparagine (N1424) in the neighbouring IIIS6 helix, thus stabilizing the channel gate in the resting state and thereby potentially hampering channel opening upon activation.

A similar effect was found for the I1306T variant, which formed stabilizing hydrogen bonds in the resting and activated states (Table 1 and Supplementary Fig. 2A–D). Only the M1425I variant in IIIS6 was different, in that it did not form stabilizing hydrogen bonds, but further increased the hydrophobicity (Table 1 and Supplementary Fig. 2E–H). Interestingly, however, this substitution strengthened the van der Waals interaction with I1306 in the neighbouring IIIS5 helix, indicating reciprocal effects within this critical interaction network in the channel gate. The hydrophobic interactions in wild-type Ca_v_3.3 are consistent with the necessary mobility of the cytoplasmic ends of the S5 and S6 helices in the gating process. On the other hand, the introduction of stabilizing interactions in the resting and activated states of the disease-associated variants is anticipated to impede both opening and closing of the gate.

### *CACNA1I* variants alter the gating properties of Ca_v_3.3 to different extents

**Figure 2:**
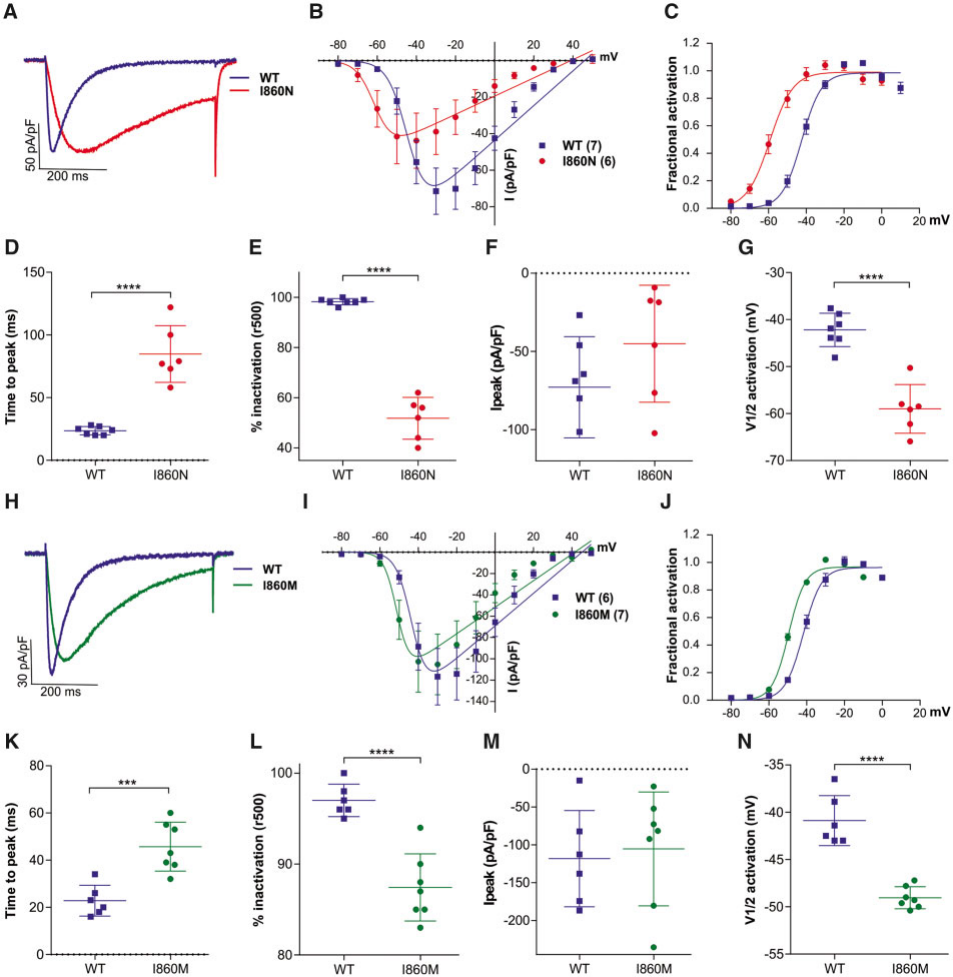
**The I860N and I860M substitutions slow current kinetics and left-shift the voltage-dependence of activation of Ca_v_3.3.** (**A**–**G**) Current properties of Ca_v_3.3-I860N (red) compared to its wild-type (WT) Ca_v_3.3 controls (blue). (**H**–**N**) Current properties of Ca_v_3.3-I860M (green) compared to its wild-type Ca_v_3.3 controls (blue). (**A** and **H**) Example maximal current traces at V_max_ with comparable current densities show slower activation and inactivation of the I860N and I860M variants. (**B** and **I**) The current-voltage relationship and (**C** and **J**) the fractional activation curves show a 15.5 mV left-shift of activation for I860N (*n* = 6) and an 8.1 mV left-shift of activation for I860M (*n* = 7), as compared to wild-type (*n* = 7 and 6, respectively). (**D** and **K**) Scatter plots of the time to peak and (**E** and **L**) the fractional inactivation after 500 ms show significantly slowed activation and inactivation of the I860N and I860M variants at V_max_. (**F** and **M**) The differences in maximum current densities (I_peak_) between WT and I860N or I860M are not significant (I860N, *P* = 0.18; I860M, *P* = 0.75). (**G** and **N**) The scatter plots of the voltage at half-maximal activation (V_**½**_) show significant hyperpolarizing shifts for I860N and I860M. Mean ± SEM; *P*-values calculated with Student’s *t*-test; ***P < 0.001, ****P < 0.0001.

To study the effects of the four disease-associated amino acid substitutions on the biophysical gating properties of the channel we performed whole-cell patch clamp recordings of HEK293T cells transfected with wild-type and mutant Ca_v_3.3 channels. In a first set of experiments we individually compared the current properties of the two substitutions of I860 with wild-type controls. The representative recordings and quantitative analyses presented in Fig. 2 and Supplementary Table 3 demonstrate that the I860N substitution caused a striking slowing of current kinetics (Fig. 2A–E), and a similar but milder effect was observed for the I860M mutant (Fig. 2H–L). At the voltage of maximal activation (V_max_) the time to peak of Ca_v_3.3-I860N was increased by more than 3-fold compared to that of wild-type Ca_v_3.3 (Fig. 2D) and ∼2-fold for Ca_v_3.3-I860M (Fig. 2K). However, analysis of the activation time constants at all test potentials revealed that for the Ca_v_3.3 mutants activation kinetics were significantly increased only at voltage steps to −50 mV and for Ca_v_3.3-I860N also at −40 mV, but not at higher test potentials (Supplementary Fig. 4). Also, both Ca_v_3.3 mutants showed a significant slowing of the inactivation kinetics with a substantially greater effect for Ca_v_3.3-I860N compared to Ca_v_3.3-I860M. During a 500-ms depolarization to V_max_ the calcium current of wild-type Ca_v_3.3 inactivated almost completely (95–98%), while inactivation of the Ca_v_3.3-I860N current only reached ∼52% (Fig. 2E) and Ca_v_3.3-I860M ∼87% (Fig. 2L). Fitting the decay phase of the current during 5-s test pulses showed significantly increased time constants for inactivation between −40 and +20 mV for Ca_v_3.3-I860N, but for Ca_v_3.3-I860M this increase does not reach significance (Supplementary Fig. 5). Furthermore, the I/V curve (Fig. 2B and I) and fractional activation plotted against the voltage of the depolarizing test pulse (Fig. 2C and J) show that the voltage dependence of activation was significantly shifted in the hyperpolarizing direction in both Ca_v_3.3 mutants. Compared to wild-type Ca_v_3.3 the Ca_v_3.3-I860N mutant resulted in a 15.5 mV left shift of the activation curve (Fig. 2G and Supplementary Table 3). The voltage dependence of activation of Ca_v_3.3-I860M was 8.1 mV left-shifted compared to wild-type Ca_v_3.3 (Fig. 2N and Supplementary Table 3).

**Figure 3:**
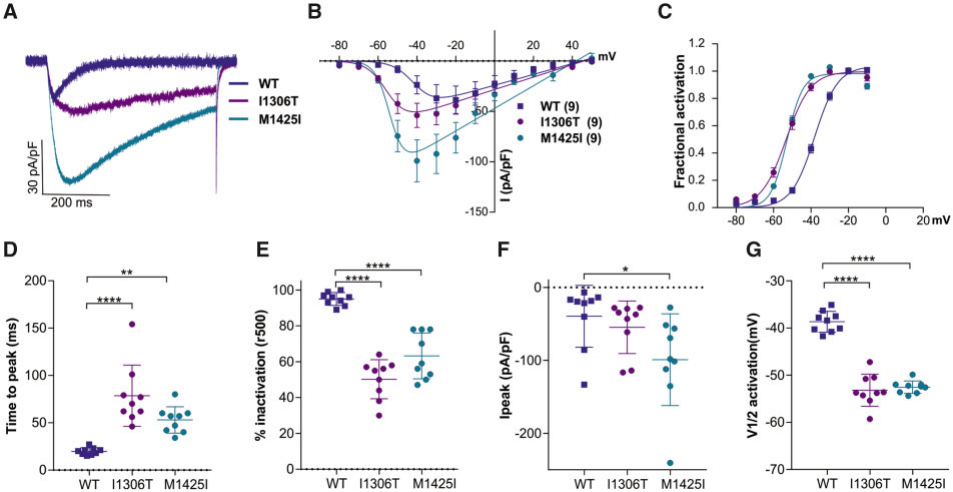
**The I1306T and M1425I substitutions slow current kinetics and left-shift the voltage-dependence of activation of Ca_v_3.3**. (**A**) Example maximal current traces of wild-type (WT) Ca_v_3.3 (blue), Ca_v_3.3-I1306T (purple) and Ca_v_3.3-M1425I (cyan) show slower activation and inactivation at V_max_. (**B**) The current-voltage relationship and (**C**) the fractional activation curves show a 6.6 mV left-shift of activation for I1306T (*n* = 9), as compared to wild-type (*n* = 9), and a 5.9 mV left-shift for M1425I (*n* = 9). (**D**) Scatter plots of the time to peak and (**E**) the fractional inactivation after 500 ms at V_max_ indicate significantly slowed activation and inactivation, respectively, of both variants. (**F**) The maximum current density (I_peak_) is doubled in M1425I (*P* = 0.03), but only insignificantly increased in I1306T (*P* = 0.73). (**G**) The scatter plot of the voltage at half-maximal activation (V_**½**_) shows a significant hyperpolarizing shift for both variants. Mean ± SEM; *P*-values calculated with ANOVA and Dunnett’s *post hoc* test; *P < 0.05, **P < 0.01, ****P < 0.0001.

Additional electrophysiological analysis of the mutants identified in Patients 5 and 6, Ca_v_3.3-I1306T and Ca_v_3.3-M1425I, demonstrated similar changes on activation and inactivation kinetics as well as a left-shifted voltage-dependence of activation, 14.5 mV and 13.7 mV, respectively. M1425I also showed an almost 3-fold increase in Ipeak (Fig. 3, Supplementary Figs 4, 5 and Supplementary Table 3).

Taken together, the current properties of the four Ca_v_3.3 mutants reveal a gain-of-function effect. The left shift of voltage dependence of activation indicates that the putative disease variants activate at voltages closer to the resting membrane potential compared to wild-type Ca_v_3.3 channels. The slowed inactivation of the mutants allows considerably more calcium to enter during depolarization. The magnitude of these effects varied between the examined Ca_v_3.3 mutants, but consistently was highest in Ca_v_3.3-I860N and lowest in Ca_v_3.3-I860M.

### Deactivation kinetics of Ca_v_3.3 channels are slowed in Ca_v_3.3-I860N, -I1306T, and -M1425I

**Figure 4:**
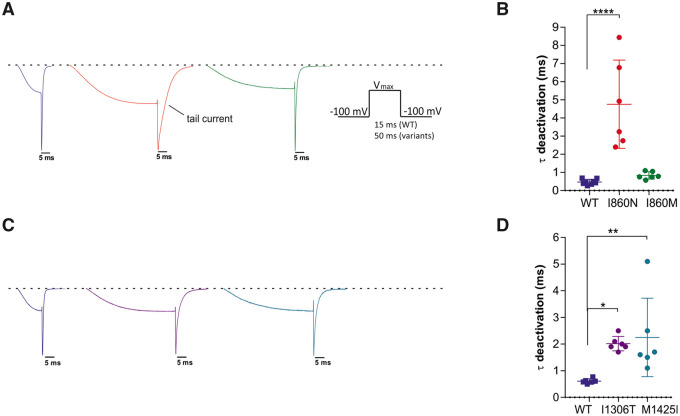
**Current deactivation is slowed in I860N, I1306T and M1425I, but not in I860M.** (**A**) Representative current traces, normalized to the peak of the tail currents of wild-type (blue), display a broadening of the tail current in I860N (red) and I860M (green) compared to wild-type. *Inset* shows the voltage-clamp protocol; voltage-steps were applied from a holding potential of −100 mV to the voltage of maximal activation for the duration necessary to achieve maximal activation without detectable inactivation [wild-type (WT) 15 ms, variants 50 ms]. (**B**) The scatter plot of the time constants (τ) of a mono-exponential fit of the decay of the tail currents demonstrates a significant slowing of deactivation in I860N versus wild-type (*P* < 0.0001), but not for I860M versus wild-type (*P* = 0.8). Wild-type (*n* = 8), I860N (*n* = 6), I860M (*n* = 6). Mean ± SEM; *P*-values calculated with ANOVA and Tukey’s *post hoc* test. ****P < 0.0001. (**C**) Representative normalized example current traces show that the tail currents of I1306T (purple) and M1425I (cyan) are broadened compared to wild-type. (**D**) The scatter plot of the time constants (τ) of a mono-exponential fit of the decay of the tail currents demonstrates a significant slowing of deactivation in I1306T versus wild-type (*P* = 0.02), as well as for M1425I versus wild-type (*P* = 0.009). *n* = 6 for all three experimental groups; *P*-values calculated with ANOVA and Dunnett’s *post hoc test*. *P < 0.05, **P < 0.01.

To examine whether the amino acid substitutions also affect the deactivation kinetics of the Ca_v_3.3 channel, we performed voltage steps to V_max_ for the duration necessary to achieve maximal activation, without detectable inactivation. As shown in Fig. 4A and C the tail current of the I860N, I1306T, and M1425I mutants were strikingly broader than those of wild-type and the I860M mutant. When fitting the decay of the tail current, we observed a substantial slowing of the time constant of deactivation in I860N as compared to wild-type Ca_v_3.3, less but still significant slowing for I1306T and M1425I, but similar deactivation kinetics for I860M and wild-type (Fig. 4B and D). The up to 10-fold slower deactivation of calcium currents in the three Ca_v_3.3 mutants suggests a considerably increased calcium influx upon repolarization as compared to both wild-type and I860M Ca_v_3.3 channels. As the speed of deactivation is a critical determinant for the oscillatory behaviour of Ca_v_3.3 channels,^10^ this channel function may be compromised in neurons expressing Ca_v_3.3-I860N, -I1306T, or -M1425I.

### Steady state inactivation and window currents are shifted closer to the resting membrane potential

**Figure 5:**
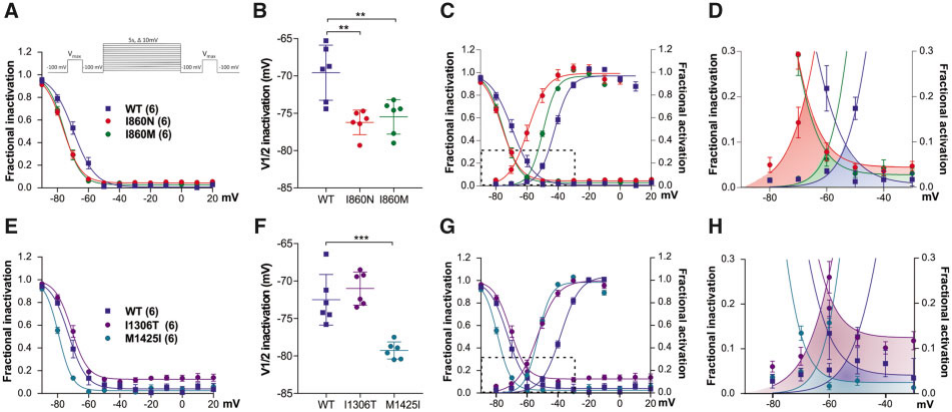
**Steady state inactivation and window currents are left-shifted in all four missense variants of Ca_v_3.3.** (**A**–**D**) Steady-state inactivation and window currents of Ca_v_3.3-I860N (red) and -I860M (green) compared to wild-type (WT) controls (blue); (**E**–**H**) the same for Ca_v_3.3-I1306T (purple) and Ca_v_3.3-M1425I (cyan). (**A**, **B**, **E** and **F**) Fractional inactivation curves and scatter plot of V_½_ of inactivation show that, compared to wild-type, the voltage-dependence of inactivation is left-shifted in I860N (6.6 mV, *P* = 0.0018), I860M (5.9 mV, *P* = 0.0045; *n* = 6), and M1425I (6.8 mV, *P* < 0.001), but not in I1306T (*P* = 0.47). The *inset* in **A** shows the steady state inactivation protocol used for these experiments. (**C** and **G**) The simultaneous display of the fractional activation and inactivation curves shows that activation of Ca_v_3.3-I860N and Ca_v_3.3-I1306T is left-shifted to greater extent than inactivation, resulting in greatly increased window currents. (**D** and **H**) Enlarged area indicated by the frames in (**C** and **G**) show the size and voltage-range of the window currents for wild-type (shaded in blue), I860N (red), I860M (green), I1306T (purple), and M1425I (cyan). Window currents of Ca_v_3.3-I860M and -M1425I are left-shifted but not enlarged. *n* = 6 for all experimental groups. Mean ± SEM; *P*-values calculated with ANOVA and Dunnett’s *post hoc* test. **P <* *0.01, ***P <* *0.001.

We performed a steady state inactivation protocol comparing the current size during test pulses before and after 5 s conditioning pulses at incrementally increasing potentials. Plotting the fractional inactivation against the voltage of the conditioning pulse revealed a significant left-shift of voltage-dependence of inactivation in all mutants except in I1306T (Fig. 5A, B, E and F). The overlapping area between activation and inactivation curves represents the window current (Fig. 5C and G). In all mutants the window current was left-shifted with peaks at voltages between −70 and −60 mV, as compared to window currents of wild-type Ca_v_3.3, which peaks near −50 mV (Fig. 5D and H). In addition, I860N and I1306T experienced a substantial increase of their window currents, because in both cases the left-shift in the voltage-dependence of activation was not accompanied by a similar left-shift in the voltage-dependence of inactivation. The voltage range of Ca_v_3.3 window current is a critical determinant of electrical activity and calcium oscillations,^10^ suggesting that the left-shifted and increased window current affects electrical activity of neurons expressing these Ca_v_3.3 variants. This further implies a persistent calcium influx close to the resting membrane potential, suggesting the possibility of calcium toxicity particular for Ca_v_3.3-I860N and Ca_v_3.3-I1306T.

### The mutant Ca_v_3.3 channels increase persistent and action potential-induced calcium currents

The observed alterations in activation, deactivation, and inactivation properties of the disease-associated Ca_v_3.3 mutants are expected to affect neuronal excitability and the magnitude of calcium influx during repetitive action potential firing. Therefore, we next examined the calcium currents in action potential clamp experiments. Action potential-like depolarizations, modelled on the shape of thalamic neurons,^43^ were repeated 99 times at 20 Hz, and currents were recorded from HEK293T cells heterologously expressing wild-type and mutant Ca_v_3.3 channels (Supplementary Fig. 3A and B). In all conditions, T-type calcium currents were facilitated during the first few action potentials and then experienced a continuous attenuation during the rest or the protocol, due to accumulating inactivation of Ca_v_3.3. Importantly, with Ca_v_3.3-I860N, -I860M, -I1306T, and -M1425I calcium currents before (persistent pace-making current), during, and after the action potential were increased by several-fold (Supplementary Fig. 3C). The increase was greatest in the early sweeps, where the persistent current of the mutants was an order of magnitude larger than that of wild-type and about twice the size for I860N and I1306T compared to I860M and M1425I (Supplementary Fig. 3D). Also, the calcium influx during the action potential was several-fold larger than in wild-type, with I860N, -I1306T and -M1425I, but not for -I860M (Supplementary Fig. 3E and F). Together, the action potential clamp recordings demonstrate that during repetitive action potential firing calcium influx is increased substantially and to different degrees in cells expressing the examined Ca_v_3.3 mutants. However, action potential clamp experiments do not account for the dynamic feedback regulation of the continually changing T-type currents onto the shape of the neuronal firing patterns.

### The mutant Ca_v_3.3 channels increase neuronal firing in a model of thalamic neurons

**Figure 6:**
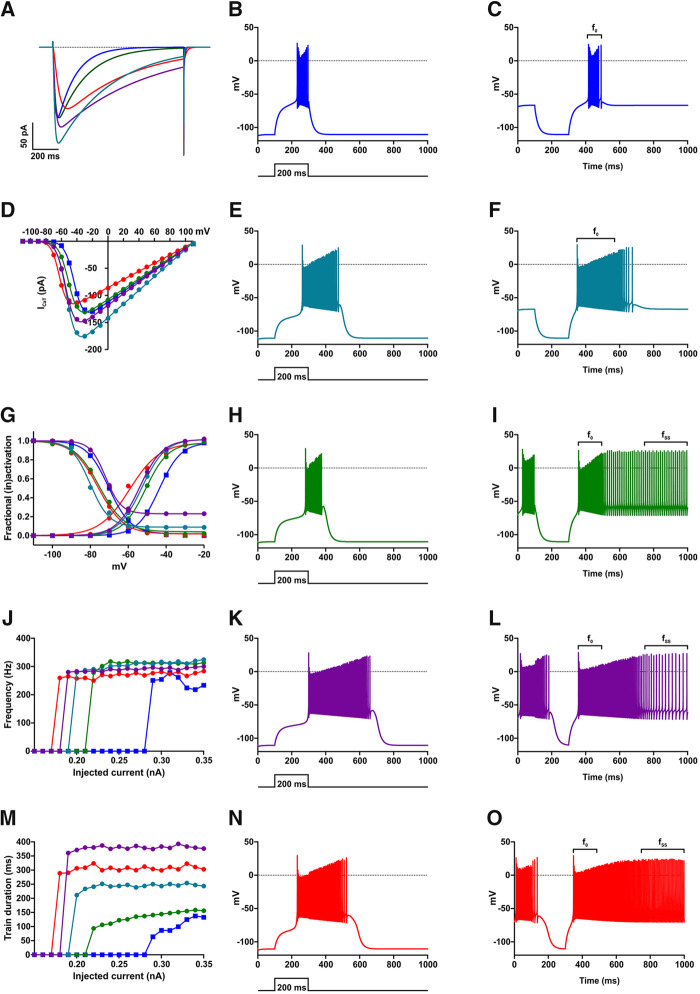
**Computer modelling of a TRN neuron shows that all amino acid substitutions increase electrical activity of Ca_v_3.3, with I860N showing a stronger effect.** The experimentally determined values for τ_activation_, τ_inactivation_, V_1/2activation_, k_act_, V_1/2inactivation_, and k_inact_ were entered in the computer model to generate I_CaT_ currents with identical kinetics (**A**) and voltage dependence of activation and inactivation (**D** and **G**) as experimentally measured (Figs 2–5) for wild-type (WT) Ca_v_3.3 (blue), M1425I (cyan), I860M (green), I1306T (purple) and I860N (red) channels. To test the effects of mutations on neuronal excitability, the resting membrane potential was set to −110 mV and 200 ms long depolarizing current pulses of increasing amplitudes were applied. (**B**, **E**, **H**, **K and N**) show the electrical activity at the minimum current injection (rheobase) necessary to stimulate an action potential. (**J**) The wild-type model needs a depolarizing pulse of 0.29 nA to initiate a train of action potentials, while M1425I fires at 0.20 nA current injection, I860M at 0.22 nA, I1306T at 0.19 nA, and I860N at 0.18 nA. (**M**) While at maximum current injection of 0.35 nA the 200_**-**_ms long pulses initiate in wild-type and I860M models an action potential train with a comparable duration (wild-type t = 132.7 ms and I860M t = 156.3 ms) the other Ca_v_3.3 mutations substantially increased the train duration (M1425I t = 243.5 ms, I1306T t = 376.3 ms, and I860N t = 302.9 ms) even at lower current injection levels. Running the models for 10 s (here shown 1 s) with the resting membrane potential unclamped showed that the wild-type model fires short action potential trains only after a brief hyperpolarizing pulse to −110 mV (**C**) with a frequency f_0_=255.1 Hz, while I860M (**I**), I1306T (**L**) and I860N (**O**) models display a continuous electrical activity, with I860M showing an initial frequency of f_0_ = 355.5 Hz and final frequency of f_SS_ = 91.5 Hz, I1306T of f_0_ = 320.9 Hz and f_SS_ = 44.9 Hz, and I860N of f_0_ = 320.3 Hz and f_SS_ = 129.2 Hz. (**F**) The M1425I model does not show a continuous electrical activity but a train with a frequency f_0_ = 338.2 Hz.

Since Ca_v_3.3 is highly expressed in thalamic neurons and these neurons are implicated in the pathophysiology of epilepsy,^17^^,^^37^^,^^44^ we simulated the effects of altered gating properties of the four Ca_v_3.3 disease-associated variants in a computer model of TRN neurons.^11^^,^^45^ In the model the parameters of the T-type calcium conductance were adjusted to closely resemble the current properties determined in the heterologous cell system (Fig. 6A, D and G; *cf*. Figs 2, 3 and 5). Simulation of neuronal excitability with the wild-type Ca_v_3.3 showed that depolarizing current injection triggers burst firing with a starting frequency of ∼250 Hz that is steadily decreasing until the burst ends abruptly at the end of the current injection (Fig. 6B and J). In simulations for the Ca_v_3.3 mutants eliciting burst firing required substantially less current injection (Fig. 6J and M) indicating that the gating defects in the Ca_v_3.3 mutants lower the neuron’s rheobase. The firing frequency was somewhat elevated (up to 300 Hz), but strikingly the burst duration was prolonged, exceeding the duration of the depolarizing current injection (Fig. 6 B, E, H, K, M and N). These effects on rheobase and burst duration were strongest for I860N and weakest for I860M (Fig. 6J and M).

When the simulations were run from a membrane potential of −60 mV, corresponding to the up-state of thalamic neurons, in the wild-type model hyperpolarizing steps to −110 mV were followed by transient burst lasting ∼100 ms (Fig. 6C). In contrast, in simulations of the mutants the bursts were lengthened, stopping spontaneously only with Ca_v_3.3-M1425I. For Ca_v_3.3-I860M, -I860N, and -I1306T bursting started at high frequency for ∼100 ms and then switched to a steady firing pattern at reduced frequency continuing until the end of the 10-s recording (Fig. 6F, I, L and O). Together, the data from the computer simulations of TRN neurons indicate that the altered gating properties of the Ca_v_3.3 mutants cause a neuronal gain-of-function effect with severe hyper-excitability.

### Expression of Ca_v_3.3-I860N and -I860M variants in mouse chromaffin cells shifts their firing mode

**Figure 7:**
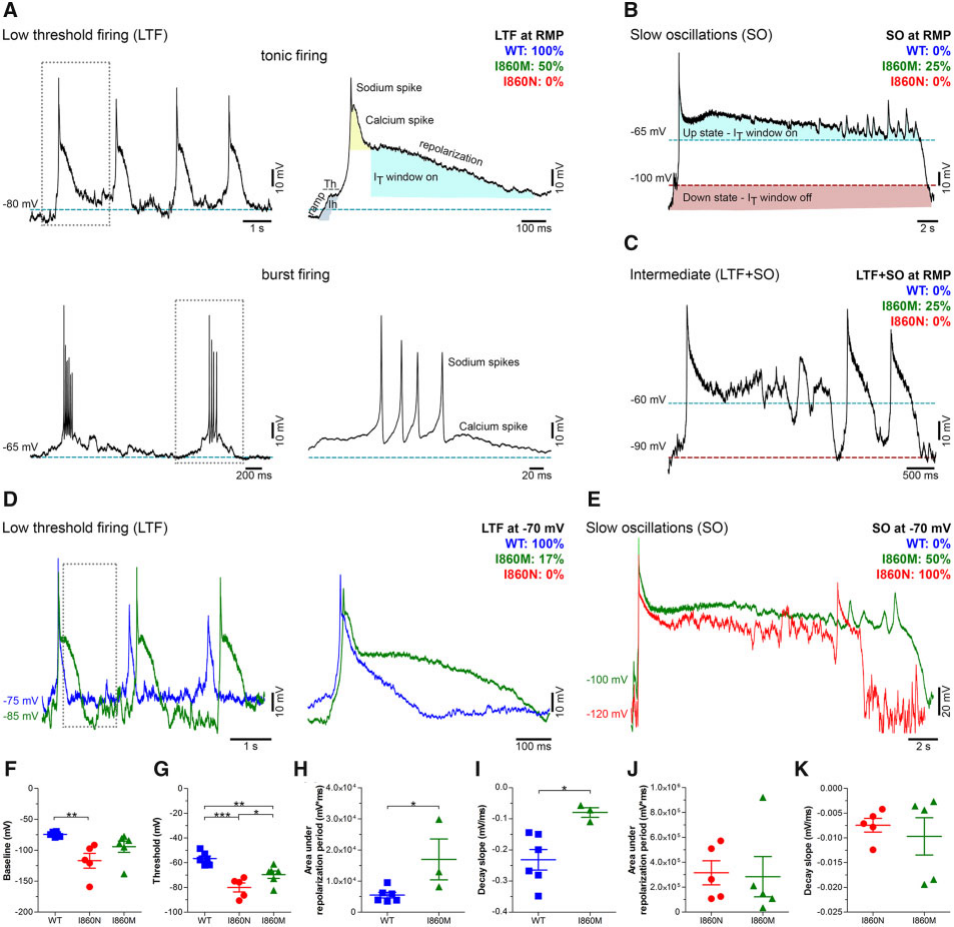
**Wild-type, I860M and I860N Ca_v_3.3 channels differently shape firing patterns of mouse chromaffin cells.** (**A**–**C**) Representative current-clamp recordings of isolated mouse chromaffin cells (MCCs) at resting membrane potential (RMP, 0 pA current injection). MCCs expressing wild-type (WT) Ca_v_3.3, Ca_v_3.3-I860M or Ca_v_3.3-I860N exhibit distinct firing modes depending on the contribution of T-type window currents (I_T_ window) and hyperpolarization-activated sodium conductances (Ih)^4^: low threshold tonic or burst firing (LTF, **A**), slow oscillations (SO, **B**) resulting from membrane potential bistability, and intermediate (LTF+SO, **C**). The percentages of MCCs displaying the different firing modes for wild-type (blue), I860M (green) and I860N (red) are indicated in each panel. Note that all firing cells expressing wild-type channels show LTF, while I860M shifts firing more towards SO (25% SO and 25% LTF+SO; wild-type *n* = 4, I860M n = 4). MCCs expressing the I860N variant display no spontaneous activity (*n* = 11). Hatched lines indicate the membrane potential at which I_T_ window current is on (blue) or off (red). (**D** and **E**) Representative current-clamp recordings after clamping the resting membrane potential to −70 mV. All firing cells expressing wild-type Ca_v_3.3 show LTF (**D**). I860M shifts firing more towards SO [17% LTF (**D**), 50% SO and 33% LTF+SO (**E**)]. MCCs expressing I860N display only SO (**E**) (*P* = 0.001) (wild-type *n* = 6, I860N *n* = 5, I860M *n* = 6). (**F**) Expression of I860N channel in MCCs hyperpolarizes the membrane potential to −116.9 ± 11.9 mV (*P* = 0.01) compared to −74.3 ± 1.5 mV for wild-type and −94.2 ± 9.1 mV for I860M. (**G**) While the depolarization threshold of MCCs expressing wild-type Ca_v_3.3 channels is −56.7 ± 2.1 mV, expression of Ca_v_3.3-I860N shifts the depolarization threshold to −79.9 ± 3.6 mV (*P* < 0.001) and Cav3.3-I860M to −69.6 ± 2.9 mV (*P* = 0.01). (**H**) The area under the action potential plateau phase is significantly higher (*P* < 0.05) for MCCs expressing I860M (16 940 ± 6562 mV × ms) compared to wild-type (5471 ± 926.8 mV × ms) when the cells show LTF activity, while it is similar for MCCs expressing I860M or I860N in the SO firing mode (**J**). The action potential decay slope (between 90% and 10%) was significantly increased in MCCs expressing I860M (−0.08 ± 0.01 mV/ms, *P* = 0.02) compared to wild-type (−0.23 ± 0.03 mV/ms) (**I**), but was similar for cells expressing I860M or I860N (**K**). Chi-square test, *t*-test or one-way ANOVA with Holm-Sidak *post hoc test*. *P <* *0.05, **P <* *0.01, ***P <* *0.001.

Finally, to examine the effects of the altered gating properties of the putative Ca_v_3.3 disease variants on the firing patterns in native excitable cells, we overexpressed wild-type Ca_v_3.3 and two representative mutants in freshly prepared mouse chromaffin cells, an established model for studying neuron-like action potential firing.^46^^,^^47^ We chose Ca_v_3.3-I860N and -I860M, because structurally they are substitutions of the same residue and functionally represent the two extremes of the observed effects on gating properties and excitability. Consistent with the critical role of Ca_v_3 channels in regulating neuronal excitability,^4^^,^^44^ and as predicted by our neuronal simulation, expression of wild-type Ca_v_3.3 in mouse chromaffin cells induced low-threshold firing (either tonic or repetitive burst-firing) (Fig. 7A). Presumably, activation of the T-type calcium current depolarizes the membrane potential to above the threshold of the sodium spikes before its gradual inactivation terminates the burst and allows the repolarization of the membrane potential.^4^ As long as inactivation of Ca_v_3.3 is incomplete the cell remains in the up-state with the membrane potential oscillating in the range of Ca_v_3.3’s window current near −50 mV. Only after the membrane potential had returned to below the lower threshold of the window current the cell would fire spikes and bursts again. This firing behaviour observed in mouse chromaffin cells expressing wild-type Ca_v_3.3 is reminiscent of the firing pattern of thalamic neurons,^4^^,^^37^ therefore, chromaffin cells represent a useful model system for studying the effects of altered T-type calcium currents on thalamic neuron-like burst firing.

When the Ca_v_3.3-I860M variant was expressed in chromaffin cells the decay of low-threshold spikes was significantly delayed, resulting in a prolonged calcium spike (Fig. 7D) or in persistent low-frequency oscillations in the up-state (Fig. 7B and E). In variance to what was predicted by the neuronal computer model, this did not result in prolonged spike firing but in occasional spikes in some of the recorded chromaffin cells (Fig. 7B, C and E). Either the oscillations mostly remained below the threshold of the sodium spikes or the incomplete recovery from inactivation of the sodium channels prevented the generation of repetitive sodium spikes. Eventually the membrane potential returned to the down-state and the cells were ready for firing again. The delayed repolarization and the resulting prolonged slow oscillation are consistent with the reduced rate of inactivation kinetics and the left-shifted window current of the Ca_v_3.3-I860M channel observed in the patch-clamp analysis. When the Ca_v_3.3-I860N variant was expressed in chromaffin cells, spike-firing ceased completely, and all recorded cells remained in the slow oscillation mode (Fig. 7E). Apparently, because of the substantially slowed T-type current inactivation and severely left-shifted and enlarged window current, cells expressing this disease-associated Ca_v_3.3 variant mostly persist in the up-state showing low-frequency oscillations without sodium spikes.

The shift in firing modes from low-threshold firing to slow oscillations in the two Ca_v_3.3 mutants was accompanied by a hyperpolarization of the baseline membrane potential (Fig. 7F) and a significant reduction of the firing threshold of the calcium spike (Fig. 7G). This is consistent with the notion that the left-shifted voltage dependence of activation in Ca_v_3.3-I860M and -I860N results in hyper-excitability of the cell. Moreover, the delayed hyperpolarization and persistent slow oscillations cause a substantially increased and potentially harmful calcium influx in cells expressing Ca_v_3.3-I860M and -I860N (Fig. 7H–K). This notion was examined by monitoring the seal stability during the recording. While initial seal quality of chromaffin cells expressing I860M or I860N was good and comparable to that of wild-type cells, after action potential firing ∼80% of I860N and 9% of I860M cells displayed leaky and unstable seal properties (Supplementary Fig. 6). This is indicative of increased cellular stress experienced, probably due to the increased calcium influx through the mutant channels.

## Discussion

In this study we report heterozygous gain-of-function mutations in *CACNA1I* associated with a congenital neurodevelopmental disorder of variable severity in three unrelated patients and one family. Patients 2–4 belonging to one family share the heterozygous *CACNA1I* missense variant p.(Ile860Met) and have borderline intellectual functioning or mild or moderate intellectual disability as main clinical feature. Patient 2 developed late-onset seizures. The other three patients (Patients 1, 5 and 6) show a severe neurodevelopmental disorder and share severe global developmental delay, absence of speech, gross motor delay, muscular hypotonia, early-onset seizures, cortical visual impairment, and feeding difficulties. Variable clinical features include various brain malformations, startle response or seizures, postnatal growth retardation, gastroesophageal reflux, and gastrostomy. The *CACNA1I* variants p.(Ile860Asn) and p.(Ile1306Thr) were *de novo* in Patients 1 and 5, respectively, while Patient 6 carried the heterozygous p.(Met1425Ile) variant absent in his mother.

The four different *CACNA1I* missense variants reported here affect amino acid residues in a known hot-spot region for gain-of-function calcium channel mutations in or next to the channel activation gate (Supplementary Fig. 1).^11–13^ Three of the four variants affect an amino acid residue located at the cytoplasmic end of an S6 helix; thus classifying the related disease as S6 calcium channelopathy.^6^ Methionine 1425 in Ca_v_3.3 corresponds to methionine 1531 in Ca_v_3.1 and to methionine 1549 in Ca_v_3.2. The *de novo CACNA1G* variant p.(Met1531Val) has recently been reported in a patient with severe epileptic encephalopathy and global cerebellar atrophy,^11^ and the heterozygous *CACNA1H* variants p.(Met1549Val) and p.(Met1549Ile) have been associated with early-onset hypertension and hyperaldosteronism with incomplete penetrance.^12^^,^^13^ Electrophysiological studies of the respective Ca_v_3 mutant channels provided evidence for a gain-of-function effect of all three mutations changing the methionine in the highly conserved MFV sequence in the Ca_v_3 IIIS6 segment.^11–13^ The recurrently mutated alanine 961 in Ca_v_3.1 [p.(Ala961Thr)], underlying severe developmental delay, cerebellar atrophy with or without epilepsy,^11^ is located next to the isoleucine corresponding to Ile860 in Ca_v_3.3 which is mutated in Patient 1 and Family 2 reported here (Supplementary Fig. 1). The Ca_v_3.1 A961T mutant showed slowed activation and inactivation kinetics and a negative shift in the steady-state activations, similar to the M1531V mutant, confirming both as gain-of-function mutations.^11^ For isoleucine 1306 substituted by threonine in Ca_v_3.3, no equivalent pathogenic germline mutation could be identified in one of the other Ca_v_ channels (Supplementary Fig. 1). However, somatic mutations resulting in the corresponding substitutions I1430T in Ca_v_3.2 (*CACNA1H*) and I1015S/V in Ca_v_1.3 (*CACNA1D*) were identified in aldosterone-producing adenomas.^48^^,^^49^ Moreover, substitutions of two neighbouring amino acid residues, such as serine 1373 in Ca_v_2.1 (*CACNA1A*) and arginine 998 in Ca_v_1.4 (*CACNA1F*), are disease-associated (Supplementary Fig. 1), but likely cause loss- rather than gain-of-function.^50–52^

Electrophysiological analysis of the Ca_v_3.3 variants revealed a gain-of-function effect on channel gating with a substantially increased calcium influx at rest and during action potential firing. These altered channel properties resulted in hyper-excitability in a model of TRN neurons. When ectopically expressed in mouse chromaffin cells, the two representative Ca_v_3.3 variants I860M and I860N which are associated with a relatively mild and severe neurological phenotype, respectively, caused a shift of firing modes from burst firing to slow oscillation. Importantly, the magnitude of all functional changes determined experimentally was consistently lower for the Ca_v_3.3-I860M variant than for Ca_v_3.3-I860N and -I1306T corresponding well to the relatively mild phenotype in patients with the I860M variant. Together, our data add *CACNA1I* to the list of disease genes associated with motor and cognitive impairment with or without epilepsy.

All four disease-associated Ca_v_3.3 variants show substantial gain-of-function effects on the gating properties. The up to 16 mV shift in the voltage-dependence of activation to hyperpolarizing potentials is the likely cause of the observed increased excitability of neurons expressing the Ca_v_3.3 channel variants. This notion is supported by the reduced threshold and increased frequency and duration of firing in the model of TRN neurons. Moreover, in the case of Ca_v_3.3-I860M and -I860N, it is evident in the lower threshold for calcium spikes recorded in mouse chromaffin cells. As TRN neurons are implicated in the generation of abnormal rhythms associated with absence epilepsy,^37^ hyper-excitability of the Ca_v_3.3 mutant channel expressed in thalamic neurons represents a plausible cause of the epileptic phenotype in the three patients with the p.(Ile860Asn), p.(Ile1306Thr) and p.(Met1425Ile) variants. Coherent with this interpretation, increased T-type calcium currents have previously been observed in TRN neurons of a genetic rat model of generalized epilepsy.^9^^,^^17^

The Ca_v_3.3 mutants showed reduced activation, deactivation, and inactivation kinetics. Slowed activation would represent a loss-of-function, but this effect was only significant at the most negative test potentials. However, the significantly slowed deactivation and inactivation kinetics will result in prolonged channel openings and increased calcium influx in active neurons, and thus represents a gain of calcium channel function. Action potential clamp experiments indicated that during physiological depolarization patterns the effects on voltage-dependence of inactivation dominate and cause substantially increased calcium influx before, during, and after the neuronal action potential. Slowed inactivation, in part also combined with altered voltage-dependence of activation and inactivation, have previously been described for disease-causing mutations in *CACNA1G* and *CACNA1H* encoding the T-type calcium channels Ca_v_3.1 and Ca_v_3.2, respectively,^11^^,^^12^ as well as in L-, PQ-, and R-type calcium channels.^53–57^ In all these genes, mutations affect highly conserved amino acid residues at the cytoplasmic end of the S6 helices, which line the channel gate. The associated phenotypes comprise neurodevelopmental anomalies and/or epileptic encephalopathy in the majority of cases. Thus, there is substantial evidence indicating that increased calcium influx because of a delayed inactivation causes a congenital neurodevelopmental disorder. Our present study adds another member of the voltage-gated calcium channel family to the S6 channelopathies, all of which probably share common pathophysiological mechanisms.

Because of their specific biophysical properties, with activation at low membrane potentials, T-type calcium channels support calcium influx at rest. The partial overlap of the activation and inactivation curves indicates the voltage range where a fraction of channels becomes activated but not yet inactivated, called the window current. In cells expressing T-type channels this causes a bi-stable resting potential and cells in the up-state generate slow, calcium-dependent oscillations of the membrane potential at about −50 mV.^10^ Particularly in the Ca_v_3.3-I860N and -I1306T variants the greater left-shift of the voltage-dependence of activation compared to that of inactivation causes a substantial left-shift and enlargement of the window current. This most probably causes the complete switch from the low-threshold firing mode to the slow oscillation mode observed in mouse chromaffin cells expressing Ca_v_3.3-I860N. As this mode is accompanied by a constant influx of calcium it will increase the likelihood of calcium toxicity and consequently may lead to aberrant development and death of neurons.^58^ We propose that these neuronal cell abnormalities represent a major cause of the severe neurodevelopmental defects observed in Patients 1, 5 and 6.

The modest and profound electrophysiological alterations observed in the Ca_v_3.3-I860M and -I860N channel variants, respectively, strikingly demonstrate that the properties of the substituted amino acid rather than the position or nature of the changed amino acid residue determine the severity of the gating defects and the clinical phenotype. At the molecular level our structure models of wild-type and mutant Ca_v_3.3 provide a possible mechanistic explanation for the left-shifted voltage-dependence of activation and slowed inactivation, as well as for the different severity of the effects in the Ca_v_3.3-I860M and -I860N channel variants. In the wild-type situation Ca_v_3.3 I860 interacts with its environment through multiple weak hydrophobic interactions. This endows the cytoplasmic end of the IIS6 helix with the necessary flexibility for its radial motion upon opening and closing of the channel gate. In the Ca_v_3.3-I860N and -I860M disease variants the substituted amino acids form hydrogen bonds with neighbouring residues, both in the activated and resting state. Stabilization of both states by addition of hydrogen bonds can easily be envisaged to slow the kinetics and left-shift activation, by slowing the state transitions and stabilizing the activated state, respectively. The addition of stabilizing interactions by the substituted amino acids, rather than the removal of a critical property of the original amino acid, is consistent with the observed dominant gain-of-function effects and also explains how two distinct substitutions at the same position can result in different disease severity. By forming two classical hydrogen bonds the stability of Ca_v_3.3-I860N substantially exceeds that of Ca_v_3.3-I860M, which only forms a single sulphur hydrogen bond. This difference in the strength of the immobilizing interactions corresponds well with the differences in the experimentally determined effects on the voltage-dependence of activation and the kinetics of deactivation and inactivation. Consistent with this notion, also the Ca_v_3.3-I1306T variant forms a hydrogen bond in an otherwise hydrophobic position. However, Ca_v_3.3-M1425I does not form hydrogen bonds; instead the substitution increases the hydrophobicity and probable van der Waals interactions with neighbouring I1306, thus further highlighting the importance of the molecular interactions of these amino acids at the cytoplasmic ends of IIIS5 and IIIS6 for proper channel gating.

Together, our functional analysis of the *CACNA1I* variants linked to neurodevelopmental disorders provide evidence for two parallel pathomechanisms contributing to the aetiology of the epileptic phenotype. First, from early development on calcium toxicity in neurons expressing the Ca_v_3.3 variants with a more severe gain-of-function effect may lead to aberrant development and loss of neurons, structural brain changes, global developmental delay, and seizures.^58^ Second, because of the specific function of Ca_v_3.3 in regulating neuronal firing patterns in the thalamus,^1^^,^^4^ defects in the channel gating properties observed in the Ca_v_3.3 variants are expected to result in hyper-excitability and firing mode shift in thalamic neurons and thus may directly contribute to epileptic seizures. At present it remains uncertain to what extent deficiencies in early neuronal development and acutely altered excitability contribute to hyper-excitability on the network level and consequently to seizures. An involvement of glia cells, which also express Ca_v_3.3, cannot be excluded.^5^^,^^58^ Nevertheless, the identification of *CACNA1I* gain-of-function mutations to be causative for a range of neurodevelopmental phenotypes, including difficult-to-treat epilepsy, makes Ca_v_3.3 an attractive target for pharmacological treatment with licensed T-type channel blockers, as well as for the development of new Ca_v_3.3-specific inhibitors.^7^ As a case in point, targeted treatment of Patient 1 with the non-selective T-type channel blocker ethosuximide resulted in improved control of seizures, in particular in a reduction of myoclonic seizures. Further studies are required to establish a target therapy of T-type calcium channel blockers in patients with this new type of S6 calcium channelopathy.

## Supplementary Material

awab101_Supplementary_Data

## Abbreviations

TRN: thalamic reticular nucleus
