# Supplementary material for: CACNA1I gain-of-function mutations differentially affect channel gating and cause neurodevelopmental disorders
Source: Brain. 2021 Mar 11;144(7):2092–106. doi: 10.1093/brain/awab101 (PMC8422349; doi:10.1093/brain/awab101)
Supplement: awab101_Supplementary_Data [file awab101_Supplementary_Data.pdf]

Supplementary Material

Whole-exome sequencing (WES), sequence data analysis and variant validation

Patient 1

Whole-exome sequencing (WES) was performed on genomic DNA extracted from leukocytes of the patient and healthy parents by CeGaT (Tübingen, Germany). Enrichment was carried out using the SureSelect Human All Exon V6 kit (Agilent). Each captured library was loaded and sequenced on the HiSeq platform (Illumina). Sequence reads were aligned to the human reference assembly (UCSC hg19) using the Burrows Wheeler Aligner (BWA mem, v0.7.17-r1188) (Li and Durbin, 2009). Variant discovery was performed with the Genome Analysis Toolkit (GATK, v3.8) (McKenna *et al.*, 2010) following the best practice recommendations of the developers. Afterwards, variants were functionally annotated and compared to those documented in publicly accessible genetic variant databases (e.g. dbSNP138, ClinVar, ExAC, and gnomAD) using AnnoVar (v2018-04-16) (Wang *et al.*, 2010). Only exonic and intronic variants that were private (absent in public database), rare (with a minor allele frequency [MAF] of ≤0.1% and no homozygotes in public databases) and located at exon-intron boundaries ranging from -10 to +10 were retained. These variants should be absent (*de novo* in patient) or present in the heterozygous state in the healthy parents (homozygous or compound heterozygous in patient). Exonic and splice variants were then prioritized by pathogenicity assessment using assessment using multiple *in silico* tools (CADD, REVEL, M-CAP, Human Splicing Finder 3.1, NetGene2-Server, and Berkeley Drosophila Genome Project-Database) (Harms *et al.*, 2020; Schneeberger *et al.*, 2020).

Sequence validation of the *CACNA1I* variant c.2579T>A (NM_021096.3) was performed in leukocyte-derived DNA of patient 1 and parents by Sanger sequencing and confirmed the missense variant to be *de novo* in the patient.

Patients 2, 3 and 4

We performed WES in the two affected sibs and their parents. WES libraries were prepared using SeqCap EZ MedExome (Roche Sequencing, Pleasanton, CA) and sequenced on a HiSeq2500 platform. Read alignment to GRCh37 (hg19) and variant calling were done with a pipeline based on BWA-MEM0.7 and GATK 3.3.0. The median coverage of the captured target region was at least 100x. Variant annotation and prioritizing were done using Cartagenia Bench Lab NGS (Agilent Technologies) as described previously (Bouman *et al.*, 2018; Houweling *et al.*, 2019).

Patient 5

Clinical exome sequencing was performed at the Division of Genomic Diagnostics at Children's Hospital of Philadelphia using DNA extracted from peripheral blood from patient 5 and unaffected biological parents. Library preparation was performed using Agilent's SureSelect XT protocol and target enrichment with Agilent's Clinical Research Exome version 1. Sequencing was performed using an Illumina HiSeq 2500 with 100 bp paired-end reads. Alignment of sequencing reads was to the hg19 genome build. Sequencing data analysis was performed using a custom bioinformatics filtration pipeline as previously described (Gibson *et al.*, 2018).

Patient 6

Using genomic DNA from patient 6 and mother, the exonic regions and flanking splice junctions of the genome were captured the IDT xGen Exome Research Panel v1.0. Massively parallel (NextGen) sequencing was done on an Illumina system with 100 bp or greater paired-end reads. Reads were aligned to human genome build GRCh37/UCSC hg19, and analyzed for sequence variants using a custom-developed analysis tool. Additional sequencing technology and variant interpretation protocol has been previously described (Retterer *et al.*, 2016). The general assertion criteria for variant classification are publicly available on the GeneDx ClinVar submission page (http://www.ncbi.nlm.nih.gov/clinvar/submitters/26957/).

Structure modelling

Homology modeling was performed using Rosetta and MOE (Molecular Operating Environment, version 2019.08, Molecular Computing Group Inc., Montreal, Canada). Additionally, ab initio Rosetta (Rohl *et al.*, 2004) was used to generate structures for loops that were not resolved in the original Cav3.1 α_1-subunit template (Zhao *et al.*, 2019). The structures for the mutants were derived from the WT model by replacing the mutated residue and carrying out a local energy minimization using MOE. The C-terminal and N-terminal parts of each domain were capped with acetylamide (ACE) and N-methylamide to avoid perturbations by free charged functional groups. The structure model was embedded in a plasma membrane consisting of POPC (1-palmitoyl-2-oleoyl-sn-glycero-3-phosphocholine) and cholesterol in a 3:1 ratio, using the CHARMM-GUI Membrane Builder (Jo *et al.*, 2009; Lee *et al.*, 2019). Water molecules and 0.15 M KCl were included in the simulation box. Energy minimizations of WT and mutant structures in the membrane environment were performed. The topology was

generated with the LEaP tool of the AmberTools20 (Case *et al.*, 2018), using force fields for proteins and lipids, ff14SBonlysc and Lipid14 (Dickson *et al.*, 2014), respectively. The WT and mutant structures were gradually heated from 0 to 300 K, keeping the lipids fixed, and then equilibrated over 1 ns. Then molecular dynamics simulations were performed for 5 ns, with time steps of 2 fs, at 300 K and in anisotropic pressure scaling conditions. Van der Waals and short-range electrostatic interactions were cut off at 10 Å, whereas long-range electrostatics were calculated by the Particle Mesh Ewald (PME) method. MOE and Pymol (The PyMOL Molecular Graphics System, Version 2.0 Schrödinger, LLC.) was used to visualize relevant interactions and point out differences in the WT and mutant structures.

Expression plasmids

The coding sequence of the human $Ca_V3.3$ subunit (Genebank ID AF393329) was obtained from a1Ic-HE3-pcDNA3, which was a gift from Edward Perez-Reyes (Addgene plasmid # 45810; http://n2t.net/addgene:45810; RRID:Addgene_45810) (Gomora *et al.*, 2002). The coding sequence of $Ca_V3.3$ was inserted downstream of the GFP in the GFP-$Ca_V1.1$ vector (Grabner *et al.*, 1998), using *Hin*dIII and *Kpn*I, generating an intermediate construct in which the coding sequences of GFP and $Ca_V3.3$ were not in frame. In order to have the coding sequences of GFP and $Ca_V3.3$ in the same open reading frame, a *Sal*I site was introduced directly in front of the initiating ATG of the $Ca_V3.3$ sequence by PCR. The PCR-generated fragment of $Ca_V3.3$ (nt 1–606), with the *Sal*I site before the starting codon, was then digested with *Sal*I and *Bsu*36I and ligated into the cleaved intermediate plasmid, yielding GFP-$Ca_V3.3$, in which the coding sequences of GFP and $Ca_V3.3$ were in frame. Transcription was under the control of a cytomegalovirus promoter.

The CaV3.3 mutations were introduced by SOE-PCR. Briefly the cDNA sequence of CaV3.3 (nt 527–2723 for I860N and I860M or nt 2567–4824 for I1306T and M1425I) was amplified by PCR with overlapping primers introducing each mutation in separate PCR reactions. For each mutation, the two separate PCR products were then used as templates for a final PCR reaction with flanking primers to connect the nucleotide sequences. These fragments were then digested with *Bam*HI/*Avr*II (for I860N and I860M) or with *Avr*II/*Hin*dIII (for I1306T and M1425I) and ligated into the corresponding sites of GFP-CaV3.3, yielding GFP-CaV3.3-I860N, I860M, I1306T and M1425I. Note that the numbering for the reported I1306T and M1425I mutations differ from the actual position in the $Ca_V3.3$ splice variant used to construct

the expression plasmids (I1271T and M1390I), which contains a shorter alternatively spliced exon in the I-II linker. For consistency we designated the constructs according to the numbering used for the disease variants I1306T and M1425I. Sequence integrity of all newly generated constructs was confirmed by sequencing (MWG Biotech, Martinsried, Germany).

Cell culture and transfections

HEK293T cells were cultured in Dulbecco´s modified Eagle medium (DMEM, Thermo Fisher Scientific) supplemented with 10% (v/v) fetal bovine serum (FBS; Merck) and penicillin-streptomycin (100 U/ml and 100 µg/ml, respectively; Thermo Fisher Scientific) and incubated at 37°C in a humidified atmosphere with 5% CO_2. HEK293T cells were transfected with 1 µg of plasmid DNA on the day of plating, using FuGENE-HD transfection reagent (Promega) and cultured in DMEM overnight. The cells were used for experiments on the first or second day after transfection.

Chromaffin cells from 6 to 8-week-old male mice were obtained as described previously with slight modifications (Marcantoni *et al.*, 2010; Calorio *et al.*, 2019) and transfected by electroporation with plasmid DNA using Mouse Neuron Nucleofector Kit (Cat# VAPG-1001, Lonza Group Ltd) (Courel *et al.*, 2008). To this end, the cell pellet was re-suspended in 300 µl of Supplemented Mouse Neuron Nucleofector Solution (MNNS) pre-warmed to RT. Per construct (Ca_V3.3 WT, I860M or I860N), 100 µl of the MNNS-cell suspension was used for nucleofection with 3 µg of DNA and program A-33 on Nucleofector™ 1 device (Amaxa). After nucleofection cells were immediately transferred into polystyrene tubes (Cat# 120160, Fisher Scientific) containing 500 µl of pre-conditioned RPMI 1640 medium (Cat# 42401018, Thermo Fisher Scientific) and allowed to recover for 20 min in a humidified incubator (37°C, 95% air and 5% CO_2). 100 µl of cell suspension was placed into the center of a pre-coated 35 mm culture dish (Cat# 353001, Fisher Scientific) containing 200 µl of pre-conditioned culture medium. 1-2 h after plating, culture medium was filled up to 2 ml and medium was completely changed after 3 h to get rid of debris. Cells were then maintained in a humidified incubator (37°C, 95% air and 5% CO2) and used for patch-clamp experiments within 1-2 d after plating.

Voltage-clamp experiments

In the HEK293T cells calcium currents were recorded with the whole-cell patch-clamp technique in voltage-clamp mode using an Axopatch 200A amplifier (Axon Instruments). Patch pipettes (borosilicate glass; Science Products) had resistances between 1.8 and 4.5 MΩ when filled with (mM) 135 CsCl, 1 $MgCl_2$, 10 HEPES, and 10 EGTA (pH 7.4 with CsOH). The extracellular bath solution contained (mM) 2 $CaCl_2$, 165 choline-chloride, 10 HEPES, and 1 Mg-Cl_2 (pH 7.4 with CsOH). Data acquisition and command potentials were controlled by pCLAMP software (Clampex version 10.2; Axon Instruments); analysis was performed using Clampfit 10.7 (Axon Instruments) and SigmaPlot 12.0 (SPSS Science) software. The current-voltage dependence was fitted according to

$$I=Gmax*(V-Vrev)/(1+\exp(-(V-V1/2)/k))$$

where Gmax is the maximum conductance of the L-type calcium currents, Vrev is the extrapolated reversal potential of the calcium current, V1/2 is the potential for half maximal conductance, and k is the slope. The conductance was calculated using $G = (-I * 1000)/(Vrev - V)$, and its voltage dependence was fitted according to a Boltzmann distribution:

$$G=Gmax/(1+\exp(-(V-V1/2)/k))$$

Steady-state inactivation curves were fitted using a modified Boltzmann equation:

$$G=(1 - Gmax)/(1+\exp((V - V1/2)/k))+Gmax$$

where V1/2 is the half-maximal inactivation voltage and k is the inactivation slope factor.

Current-clamp experiments

The whole cell current-clamp recordings of isolated mouse chromaffin cells were performed in perforated-patch mode. The patch pipettes had a resistance between 1.8-4 MΩ when filled with (in mM): 10 NaCl, 10 KCl, 76 K_2SO_4, 1 $MgCl_2$, 5 HEPES, adjusted to pH 7.35 with KOH and supplemented with amphotericin B (240 µg/m; Cat# Y0000005, Merck KGaA). To facilitate sealing, glass pipettes were dipped in amphotericin-free intracellular solution before being back-filled with solution containing amphotericin, which was light-protected and kept cold (0–4°C) during the experiment. The external bath solution contained (in mM): 140 NaCl, 3.6 KCl, 2 $NaHCO_3$, 0.5 NaH_2PO_4, 0.5 $MgSO_4$, 2.5 $CaCl_2$, 5 HEPES, 5 glucose, adjusted to pH 7.4 with

NaOH. Recordings were performed at room temperature (20-24 °C) using an EPC 10 amplifier (HEKA Elektronik) controlled by PatchMaster software (version 2.80, HEKA Elektronik). Data were sampled at 10 kHz and low-pass filtered at 2.9 kHz. Action potentials were recorded without injecting any current (spontaneous activity) and after clamping the resting membrane potential to -70 mV. Offline data analysis of AP parameters (Fig. 7) was performed using Clampfit software (version 10.7, Axon Instruments). Firing modes of chromaffin cells were characterized according to T-type channel mediated firing patterns of thalamic neurons (Cain and Snutch, 2010).

Computer model

Modelling was performed in the NEURON simulation environment (Hines and Carnevale, 1997) using the model for thalamic relay neurons (Destexhe *et al.*, 1996) from the model database at Yale University (https://senselab.med.yale.edu/modeldb/). The electrophysiological properties of the Cav3.3 channels were modelled using Hodgkin-Huxley equations as described previously (Huguenard and Prince, 1992; Destexhe *et al.*, 1996). The values of native T-type channels were substituted by the experimentally obtained values for the WT, I860N, I860M, I1306T and M1425I mutants.

The equations to model the WT CaV3.3 channel were:

m_inf=1/(1+exp(-(v+50)/7.4)

h_inf=1/(1+exp((v+78)/5.0)

tau_m=(12+1/(exp((v+25)/10)+exp(-(v+100)/15)))/phi_m

tau_h=(125+1.0/(exp((v+46)/4)+exp(-(v+405)/50)))/phi_h

The equations to model the CaV3.3-I860M channel were:

m_inf=1/(1+exp(-(v+57.1)/7.4))

h_inf=1/(1+exp((v+79)/7.5))

tau_m=(13+1.0/(exp((v+25)/10)+exp(-(v+100)/15)))/phi_m

tau_h=(200+1.0/(exp((v+46)/4)+exp(-(v+405)/50)))/phi_h

The equations to model the CaV3.3-I860N channel were:

m_inf=1/(1+exp(-(v+67)/6))

h_inf=1/(1+exp((v+79)/7.5))

tau_m=(30+1/(exp((v+25)/10)+exp(-(v+100)/15)))/phi_m

tau_h=(480+1/(exp((v+46)/4)+exp(-(v+405)/50)))/phi_h

The equations to model the CaV3.3-I1306T channel were:

m_inf=1/(1+exp(-(v+60)/7.4))

h_inf=1/(1+exp((v+74)/4))

tau_m=(13+1/(exp((v+25)/10)+exp(-(v+100)/15)))/phi_m

tau_h=(620+1/(exp((v+46)/4)+exp(-(v+405)/50)))/phi_h

The equations to model the CaV3.3-M1425I channel were:

m_inf=1/(1+exp(-(v+59)/7.4))

h_inf=1/(1+exp((v+84)/3))

tau_m=(9+1/(exp((v+25)/10)+exp(-(v+100)/15)))/phi_m

tau_h=(380+1/(exp((v+46)/4)+exp(-(v+405)/50)))/phi_h

Supplementary Tables

Supplementary Table S1. Clinical characteristics of patients with *CACNA1I* missense variants.

	**Patient #**	**1<br>Family 1**	**2<br>Family 2**	**3<br>Family 2**	**4<br>Family 2**	**5<br>Family 3**	**6<br>Family 4**
**Variant**	Mutation NM_021096.3 NP_066919.2	c.2579T>A p.(Ile860Asn)	c.2580C>G p.(Ile860Met)	c.2580C>G p.(Ile860Met)	c.2580C>G p.(Ile860Met)	c.3917T>C p.(Ile1306Thr)	c.4275G>A p.(Met1425Ile)
	Exon	14	14	14	14	22	25
	Origin	*de novo*	ND	Maternally inherited	Maternally inherited	*de novo*	Absent in mother, father not tested
**Evaluation**	Nationality	Caucasian	Caucasian	Caucasian	Caucasian	Caucasian	Caucasian
	Sex	Female	Female	Male	Female	Female	Male
**Pregnancy and birth**	Pregnancy	Reduced fetal movements; breech presentation	ND	Uncomplicated	Uncomplicate d	Gestational diabetes; delivery was uncomplicated	Maternal depression
	Birth at (weeks)	37+6	ND	38	42	39	34+6
	Birth weight (z-score)	3320 g (+0.41 z)	ND	ND	3000 g (-1.12 z)	4082 g (+1.44 z)	2912 g (+0.77 z)
	Birth length (z-score)	50 cm (-0.12 z)	ND	ND	ND	55 cm (+1.5 z)	48.5 cm (+0.34 z)
	OFC birth (z-score)	34.5 cm (+0.19 z)	ND	ND	ND	ND	33.5 cm (+0.32 z)
**Last examination**	Age	8 y 1 m	58 y	28 y 11 m	31 y	21 m	8 y 3 m
	Weight (z-score)	17 kg (-3.31 z)	95 kg (+2.93 z)	77.5 kg (+0.06 z)	70 kg (+1.17 z)	10 kg (-1.16 z)	20.3 kg (-2.36 z)
	Height (z-score)	112 cm (-3.07 z)	163 cm (-0.80 z)	179.5 cm (-0.17 z)	172 cm (+0.61 z)	83 cm (-0.60 z)	109 cm (-3.90 z)
	OFC (z-score)	50 cm (-1.19 z)	57 cm (+1.30 z)	58.5 (+0.99 z)	57.5 cm (+1.66 z)	46.5 cm (-1.21 z)	53.4 cm (6 y 8 m) (+0.74 z)
**Development**	DD/ID	Severe global developmental delay; minimal interaction	IQ 70-85	ID (IQ 50-70)	ID (IQ 36-51); social-emotional level: 1.5-3 y	Severe developmental delay and hypotonia without regression	Developmental impairment (understands some commands)
	Motor development	No motor milestones achieved	ND	Normal	Normal	Gross motor delay; no sitting independently; poor head control (but improving)	Walked at 8 y; partially undresses; not toilet trained
	Speech impairment	Nonverbal	ND	ND	Speech retardation	Nonverbal	Nonverbal
**Neurological features**	Muscular hypotonia	Severe truncal hypotonia; hypertonia of the lower limbs	No	No	No	Severe hypotonia	Hypotonia
	Startle reactions/ hyperexcitability	2 y: hyperexcitability; between 4 and 6 y: startle seizures triggered by noise	No	No	No	Startle response to some auditory input	No
	Seizures	Yes	Yes	No	No	Yes	Yes

	Seizure onset	4 m	58 y	NA	NA	2 wks	2 y
	Seizure type	Epileptic apnea with 4 m; later mainly epileptic myoclonus; startle seizures with myoclonic seizures followed by a tonic episode; rare grand mal seizures	NA	NA	NA	Began with clonic seizures, then became more extensor spasms; responded to phenobarbital; rare seizures at last visit (21 m)	Staring spells with behavioural arrest +/- hitting right side of head
	EEG	3 y: mild susceptibility to seizures<br>8 y: monomorphic alpha activity; intermittent theta-slowing; mild focal epileptic activity over the left hemisphere with temporal maximum; epileptic myoclonus; mild diffuse cortical dysfunction	ND	ND	ND	22 m: normal	7 y: rare, intermittent, diffuse background slowing and abundant epileptiform discharges in right and left centro-parietal regions; diffuse focal cortical dysfunction and increased risk of focal epileptogenicity
	Response to treatment	Difficult to treat; controlled to some extent by medication	Controlled on medication (Diphantoine)	NA	NA	Controlled on medication (Keppra)	Controlled on medication
	MRI scan	4 m: unremarkable; 2 y 7 m: mild frontal accentuated brain atrophy; flat brainstem; mildly delayed myelination	CT cerebrum: no abnormalities<br><br>MRI: ND	ND	ND	12 m: unremarkable	5 y: hypogenesis of the corpus callosum with tapering of the posterior body and absence of splenium; mild interdigitation of anterior frontal gyri consistent with deficiency of anterior falx; prominent perivascular spaces; slight asymmetry of hippocampi
**Other features**	Hearing	Normal to hyperacusis	Hearing loss	Normal	Subnormal	Normal	Normal
	Vision	Cortical visual impairment	Reading glasses	Amblyopia; myopia	Myopia	Cortical visual impairment	Cortical visual impairment
	Feeding	Feeding problems; pharyngeal dyskinesia; G-tube at 8 y	ND	No feeding problems	No feeding problems	Difficulty feeding issues with reflux and silent aspiration; requires G-tube	Gastroesophageal reflux; poor chewing
	Craniofacial dysmorphism	No	No	No	No	No	Open mouth with frequent drooling; full lips; large, prominent upper incisors
	Other findings	Mixed sleep apnea with central sleep apnea, hypopnea and OSAS; non-invasiv ventilation overnight; hip dysplasia on the right; hip luxation on the left	Depression	Arthritis in neonatal period	No	Poor sleep	Growth hormone deficiency

Abbreviations: CT, computed tomography; DD, developmental delay; EEG, electroencephalogram; ID, intellectual disability; IQ, intelligence quotient; G-tube, gastrostomy tube; m, months; MRI, magnetic resonance imaging; NA, not applicable; ND, no data; NSVD, normal spontaneous vaginal delivery; OFC, occipital frontal circumference; OSAS, obstructive sleep apnea syndrome; wks, weeks; y, years; z, z-score.

Supplementary Table S2. Minor allele frequency (MAF) and *in silico* pathogenicity predictions of *CACNA1I* variants identified in six individuals.

Patient(s)	Genomic Position	Nucleotide change (NM_021096.3)	Amino acid change (NP_066919.2)	MAF [%] worldwide	Pathogenicity predictions			Genetic tolerance
					CADD	REVEL	M-CAP	
**1**	22:40055832	c.2579T>A	p.(Ile860Asn)	Absent	26.8	0.878	0.436	Highly intolerant
**2 – 4**	22:40055833	c.2580C>G	p.(Ile860Met)	Absent	24.7	0.832	0.323	Highly intolerant
**5**	22:40061568	c.3917T>C	p.(Ile1306Thr)	Absent	28.1	0.983	0.719	Highly intolerant
**6**	22:40066123	c.4275G>A	p.(Met1425Ile)	Absent	26.3	0.863	0.719	Highly intolerant

The functional impact of the identified variants was predicted by the Combined Annotation Dependent Depletion (CADD) tool, the Rare Exome Variant Ensemble Learner (REVEL) scoring system, and the Mendelian Clinically Applicable Pathogenicity (M-CAP) Score. CADD is a framework that integrates multiple annotations in one metric by contrasting variants that survived natural selection with simulated mutations. Reported CADD scores are phred-like rank scores based on the rank of that variant's score among all possible single nucleotide variants of hg19, with 10 corresponding to the top 10%, 20 at the top 1%, and 30 at the top 0.1%. The larger the score the more likely the variant has deleterious effects; the score range observed here is strongly supportive of pathogenicity, with all observed variants ranking above ~99% of all variants in a typical genome and scoring similarly to variants reported in ClinVar as pathogenic (~85% of which score >15) (Kircher *et al.*, 2014). REVEL is an ensemble method predicting the pathogenicity of missense variants with a strength for distinguishing pathogenic from rare neutral variants with a score ranging from 0-1. The higher the score the more likely the variant is pathogenic (Ioannidis *et al.*, 2016). M-CAP is a classifier for rare missense variants in the human genome, which combines previous pathogenicity scores (including SIFT, Polyphen-2, and CADD), amino acid conservation features and computed scores trained on mutations linked to Mendelian diseases. The recommended pathogenicity threshold is >0.025 (Jagadeesh *et al.*, 2016). Genetic tolerance at the affected amino acid position in the protein was predicted by MetaDome (Wiel *et al.*, 2019).

Supplementary Table S3. Properties of calcium currents from Ca$_V$3.3 WT, I860N, I860M, T1306I and M1425I.

	**Ca$_V$3.3 WT**	**Ca$_V$3.3 I860N**	**p-value t-test**	**Ca$_V$3.3 WT**	**Ca$_V$3.3 I860M**	**p-value t-test**
**Ipeak (pA/pF)**	-72.9±12.2	-45.0±15.3	0.18	-118.2±26.0	-105.3±26.6	0.75
**Gmax (nS/nF)**	1095.7±204.9	585.4±205.9	0.11	1690.4±361.2	1473.1±398.7	0.71
**V1/2 act. (mV)**	-42.2±1.3	-58.7±2.0	****	-40.9±1.1	-49.1±0.4	****
**k act. (mV)**	5.0±0.1	5.9±0.6	0.17	5.4±0.9	4.6±0.2	0.39
**Vrev (mV)**	43.4±2.3	42.1±5.4	0.83	47.0±1.9	42.6±1.7	0.13
**time to peak (ms)**	23.6±1.3	84.8±9.2	****	22.8±2.7	45.7±3.7	***
**r500 (%)**	98.3±0.5	51.9±3.4	****	97.1±0.7	87.5±1.4	****
**n**	7	6	--	6	7	--
	**Ca$_V$3.3 WT**	**Ca$_V$3.3 I860N**	**p-value ANOVA**		**Ca$_V$3.3 I860M**	**p-value ANOVA**
**V1/2 inact. (mV)**	-69.6±1.2	-76.2±0.7	0.0018**	--	-75.5±0.9	0.0045**
**k inact. (mV)**	6.9±0.4	5.7±0.3	0.09	--	5.3±0.3	0.02*
**τ activation (ms)**	12.7±2.1	35.8±11.2	0.048*	--	15.6±1.2	0.9
**τ inactivation (ms)**	137.8±13.3	564.2±64.3	****	--	214.3±9.9	0.3
**τ deactivations (ms)**	0.5±0.05	4.8±1.0	****	--	0.8±0.1	0.8
**n (n deactivation)**	6(8)	6(6)	--	--	6(6)	--
	**Ca$_V$3.3 WT**	**Ca$_V$3.3 I1306T**	**p-value ANOVA**		**Ca$_V$3.3 M1425I**	**p-value ANOVA**
**Ipeak (pA/pF)**	-39.5±14.1	-54.7±12.0	0.73	--	-109.1±29.3	0.03*
**Gmax (nS/nF)**	660.8±253.4	747.8±160.7	0.95	--	1372.9±348.2	0.12
**V1/2 act. (mV)**	-38.7±0.7	-53.2±1.1	****	--	-52.4±0.6	****
**k act. (mV)**	5.9±0.5	6.4±0.5	0.59	--	4.1±0.4	0.046*
**Vrev (mV)**	40.6±4.1	42.3±3.1	0.9	--	39.6±2.8	0.94
**time to peak (ms)**	19.8±1.3	78.6±10.8	****	--	53.0±5.7	0.004**
**r500 (%)**	95.1±1.2	50.2±3.7	****	--	63.3±5.2	****
**n**	9	9	--	--	9	--
	**Ca$_V$3.3 WT**	**Ca$_V$3.3 I1306T**	**p-value ANOVA**		**Ca$_V$3.3 M1425I**	**p-value ANOVA**
**V1/2 inact. (mV)**	-72.5±1.4	-71.0±0.8	0.47	--	-79.3±0.5	***
**k inact. (mV)**	5.4±0.5	4.9±0.4	0.6	--	4.4±0.3	0.046*
**τ activation (ms)**	7.6±1.0	16.3±1.6	0.0014**	--	11.2±1.6	0.17
**τ inactivation (ms)**	79.4±6.6	690.4±36.5	****	--	419.1±24.1	****
**τ deactivation (ms)**	0.6±0.04	2.0±0.1	0.02*	--	2.2±0.6	0.009**
**n**	6	6	--	--	6	--

All data are presented as Mean ± SEM. *P*-values in the upper part of the table were calculated using the student t-test, p-values in the lower part of the table were calculated using the ANOVA and Tukey's or Dunnett's posthoc test. * $P<0.05$, ** $P<0.01$, *** $P<0.001$, **** $P<0.0001$

Supplementary Figures

	IIS6		IIIS5		IIIS6
Ca$_V$2.1 (*CACNA1A*)	706 LNVFLAIAVDNLA 718	…	1371 VNSLKNVFNILIV 1383	…	1499 PFFFVNIFVALII 1511
Ca$_V$2.2 (*CACNA1B*)	701 LNVFLAIAVDNLA 713	…	1276 VNSLKNVLNILIV 1288	…	1404 PFFFVNIFVALII 1416
Ca$_V$2.3 (*CACNA1E*)	695 LNVFLAIAVDNLA 707	…	1283 VTSLKNVFNILIV 1295	…	1411 PFFFVNIFVALII 1423
Ca$_V$1.1 (*CACNA1S*)	653 LNVFLAIAVDNLA 665	…	922 FVAISTIGNIVLV 934	…	1106 AFFMMNIFVGFVI 1118
Ca$_V$1.2 (*CACNA1C*)	745 LNVFLAIAVDNLA 757	…	1023 FVAIRTIGNIVIV 1035	…	1154 AFFMMNIFVGFVI 1166
Ca$_V$1.3 (*CACNA1D*)	764 LNVFLAIAVDNLA 776	…	1029 FVAIRTIGNIMIV 1041	…	1160 AFFMMNIFVGFVI 1172
Ca$_V$1.4 (*CACNA1F*)	750 LNVFLAIAVDNLA 762	…	994 FVAIRTIGNIMIV 1006	…	1125 AFFMMNIFVGFVI 1137
Ca$_V$3.1 (*CACNA1G*)	956 FNLLVAILVEGFQ 968	…	1406 MSSLKPIGNIVVI 1418	…	1525 AFFVLNMFVGVVV 1537
Ca$_V$3.2 (*CACNA1H*)	1007 FNLLVAILVEGFQ 1019	…	1424 ISSLRPIGNIVLI 1436	…	1543 SFFVLNMFVGVVV 1555
Ca$_V$3.3 (*CACNA1I*)	854 FNLLVA**I**LVEGFQ 866	…	1300 ISSLKP**I**GNIVLI 1312	…	1419 SFFVLN**M**FVGVVV 1431
	Ile860		Ile1306		Met1425

Supplementary Figure S1. Partial protein sequence alignments of Cav family members. Partial amino acid sequence alignment of the cytoplasmic end of transmembrane helices IIS6, IIIS5, and IIIS6 of human CaV3.3 (NP_066919.2) and homologs (CaV2.1 [NP_075461.2], CaV2.2 [NP_000709.1], CaV2.3 [NP_001192222.1], CaV1.1 [NP_000060.2], CaV1.2 [NP_000710.5], CaV1.3 [NP_000711.1], CaV1.4 [NP_005174.2], CaV3.1 [NP_061496.2], and CaV3.2 [NP_066921.2]) shows evolutionary conservation of the mutated amino acid residues Ile860, Ile1306, and Met1425 in CaV3.3 (CACNA1I; bold) within the human Cav family (shaded in gray). Amino acid residues in other CaV members, the exchange of which has been associated with human disease, are highlighted in red bold font.

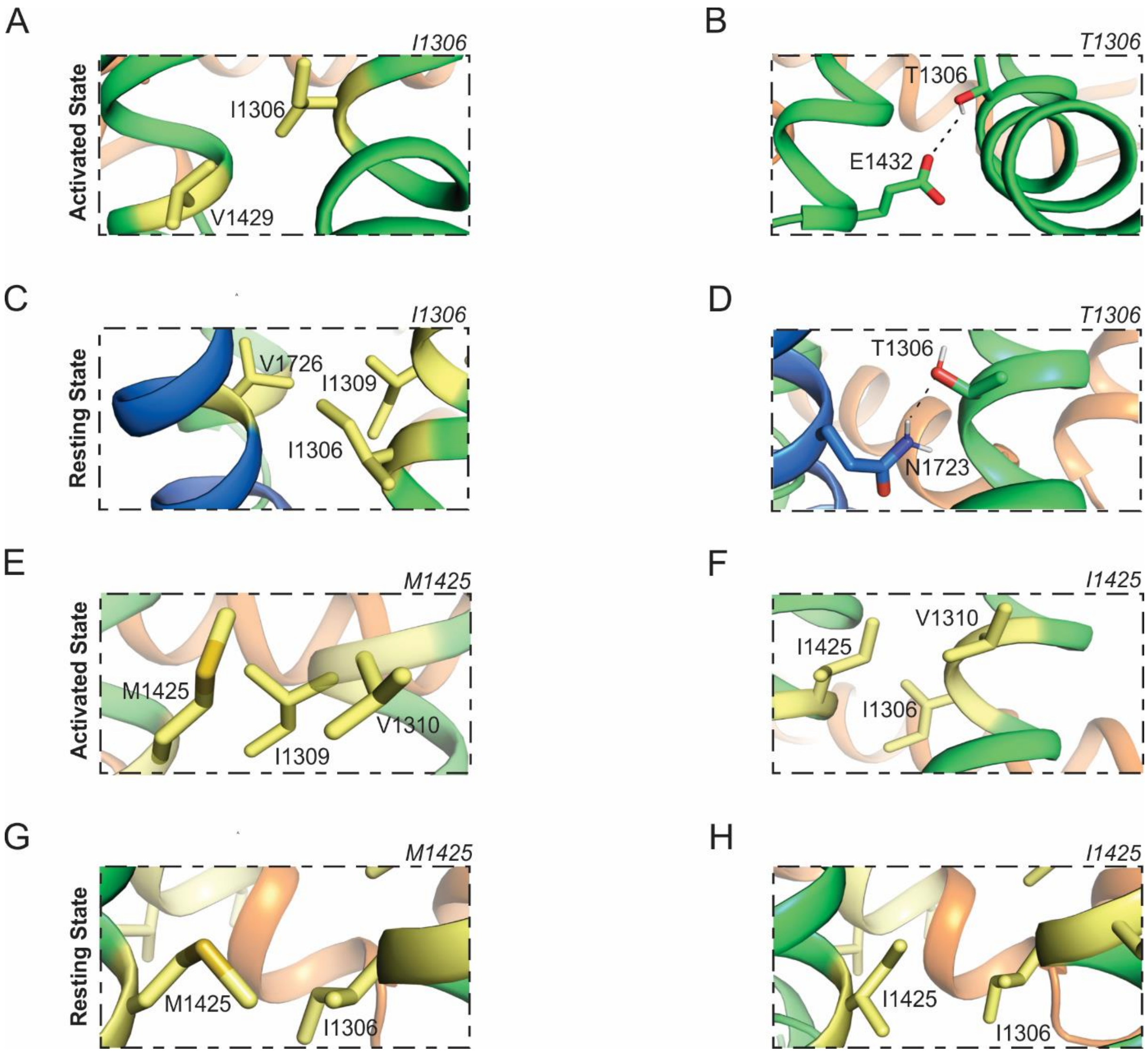

Supplementary Figure S2. Intramolecular interactions of I1306T and M1306I variants. Structure homology models of Ca$_V$3.3-I1306T and -M1425I variants highlighting the side-chain interactions of the substituted amino acids. **(A, C)** The hydrophobic wild-type I1306 in IIIS5 is not stabilized by strong interactions. In contrast, the substituted T1306 forms a hydrogen bond with E1432 **(B)** in the activated state and with N1723 **(D)** in the resting state. **(E-H)** The weakly hydrophobic wild-type M1425 in IIIS6 becomes substituted by the stronger hydrophobic isoleucine. Although this does not stabilize the residue like the hydrogen bonds formed in the other mutations **(B, D;** and cf. **Fig. 2)**, in both the activated and resting state I1425 interacts with I1306, underlining the sensitivity of these residues to changes in the strength of their reciprocal interactions.

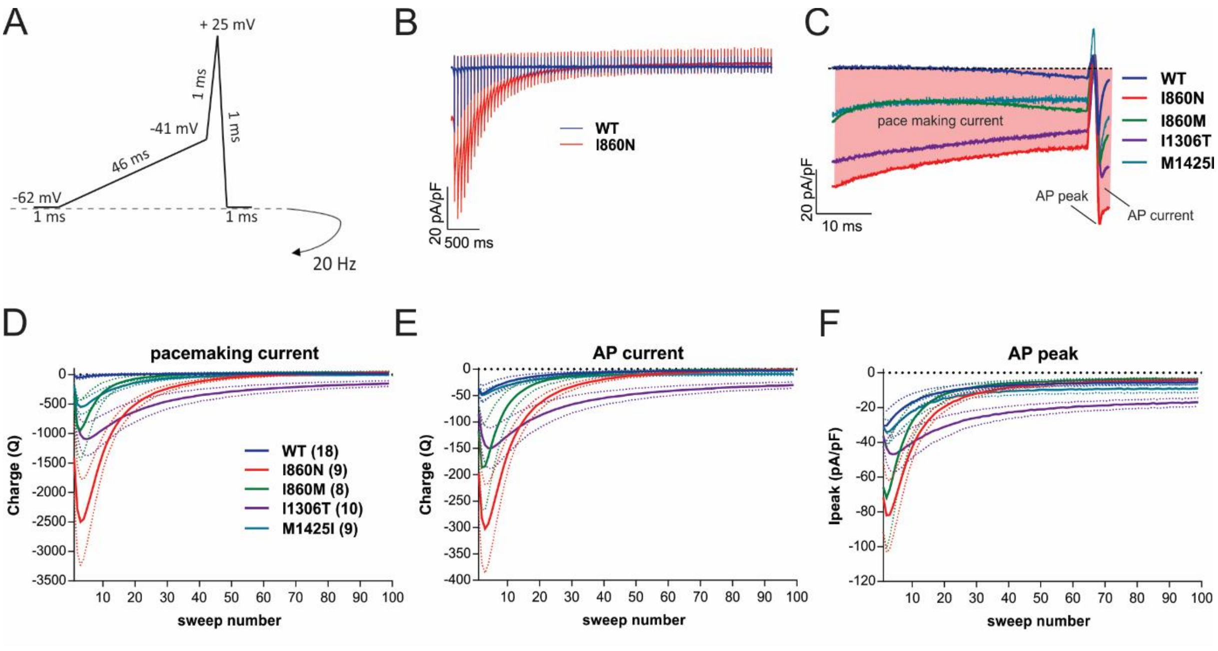

Supplementary Figure S3. AP-like voltage-clamp recordings indicate an increase in persistent pace-making and AP current in all four Ca$_V$3.3 variants. (A) AP-shaped stimulation protocol designed according to modeled burst activity of a thalamic neuron (Cazade *et al.*, 2017). The AP-clamp protocol contained 99 sweeps at 20 Hz. **(B)** Example traces of WT Ca$_V$3.3 (blue) and Ca$_V$3.3-I860N (red) as a continuous display of 99 sweeps; for clarity only the variant with the highest increase is shown. **(C)** Example traces of one single AP at the beginning of the train, for WT (blue), I860N (red), I860M (green), I1306T (purple), and M1425I (cyan). The overlay shows the increase in pace making current, AP current (both shaded in red), and AP peak current amplitude. **(D-F)** Show the average integrals (±SEM, indicated as dotted line above and below each line graph) of the pace making current **(D)**, the AP current **(E),** and the AP peak amplitude **(F)** for each AP during the entire protocol (x-axis) of WT (blue, n=18), I860N (red, n=9), I860M (green, n=8), I1306T (purple, n=10), and M1425I (cyan, n=9). The differences for all three parameters are much bigger at the beginning of the AP-train, peaking at sweep number 3, than at the end when the AP's have become attenuated. **(D)** While there is nearly no pace-making current in WT, M1425I shows a pace-making current of up to -550±125 nC/µF, I860M up to -944±504 nC/µF, I1306T up to -1020±249 nC/µF and I860N even up to -2500±736 nC/µF, at AP 3. **(E)** At AP 3, the AP current of WT (-45±15 nC/µF) and M1425I (-48±10 nC/µF) are similar but the AP current in I1306T (-144±36 nC/µF), I860M (-185±82 nC/µF) and I860N (-302±84 nC/µF) show a 3-, 4-, and 7-fold increase, respectively. **(F)** The peak at AP 3 shows a 3- to 4-fold increase for I860M and I860N and only a small increase in I1306T and M1425I.

A

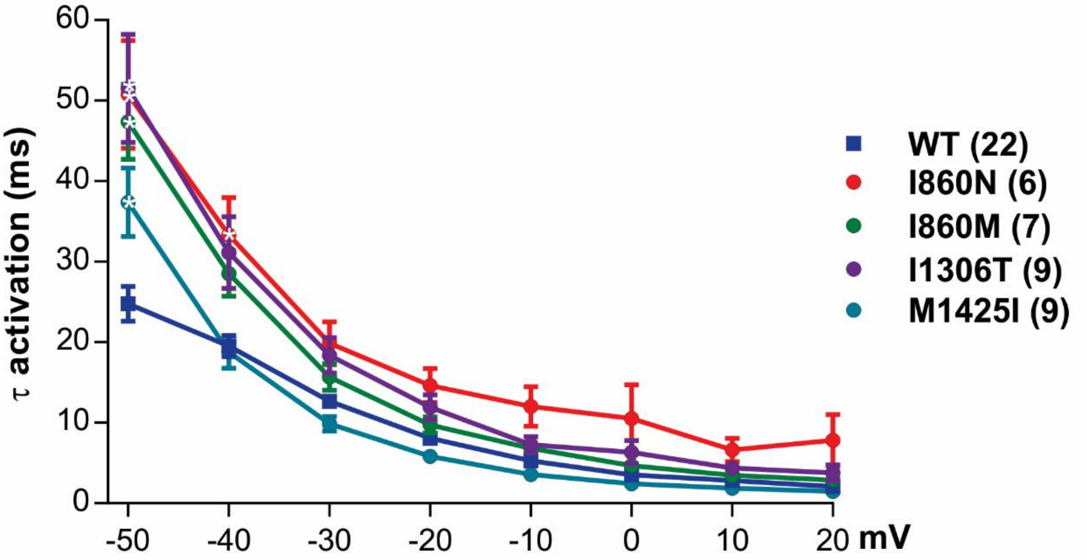

B

	P-value			
Comparison at V	WT vs. I860N	WT vs. I860M	WT vs. I1306T	WT vs. M1425I
-50 mV	***	***	***	0.02*
-40 mV	0.009**	0.34	0.38	0.34
-30 mV	0.17	0.5	0.56	0.7
-20 mV	0.23	0.66	0.64	0.8
-10 mV	0.25	0.72	0.84	0.93
0 mV	0.18	0.78	0.81	0.9
10 mV	0.72	0.97	0.94	0.84
20 mV	0.39	0.82	0.91	0.97

Supplementary Figure S4. I860N, I860M, I1306T and M1425I show slowed activation kinetics at -50 mV. Time constants of activation were calculated from fits of the rising current phase during 500 ms step depolarizations to the indicated voltages. **(A, B)** At -50 mV all four mutants show a significant slowing of T_{act}. At -40 mV only I860N shows a significant slower kinetics of activation. At higher depolarizations none of the T_{act} values were significantly different from wild-type. Mean±SEM; *P*-values calculated with repeated measures ANOVA and the Holm-Sidak test for multiple comparisons.

A

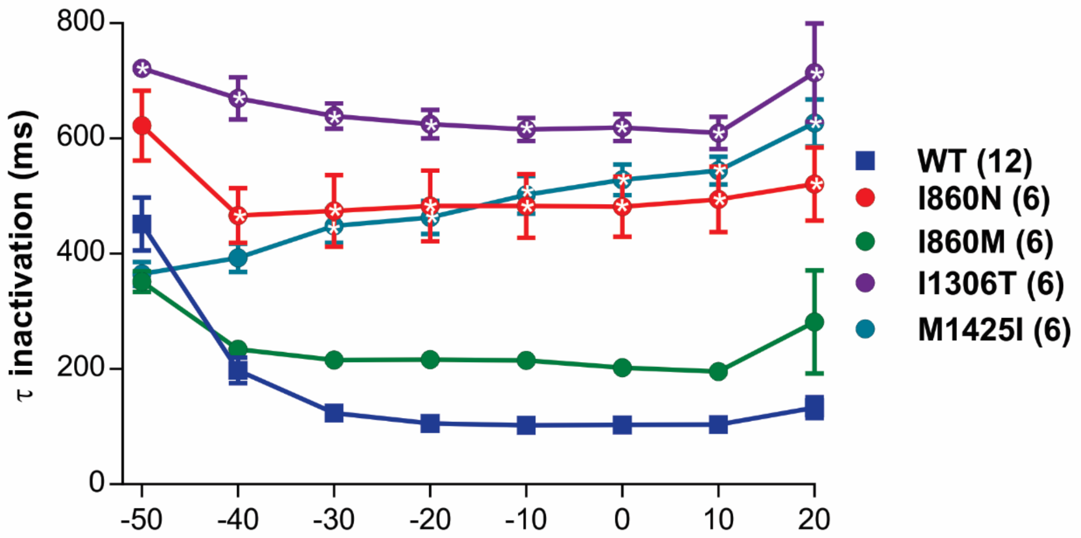

B

	P-value			
**Comparison at V**	**WT vs. I860N**	**WT vs. I860M**	**WT vs. I1306T**	**WT vs. M1425I**
**-50 mV**	0.16	0.23	0.046*	0.22
**-40 mV**	0.03*	0.91	***	0.16
**-30 mV**	***	0.45	***	0.002**
**-20 mV**	***	0.31	***	***
**-10 mV**	***	0.3	***	***
**0 mV**	***	0.41	***	***
**10 mV**	***	0.41	***	***
**20 mV**	***	0.09	***	***

Supplementary Figure S5. All four mutants show slowed inactivation kinetics. The time constants of activation (T_{inact}) were determined by fitting the decay phase of currents during 5 s depolarizations to the indicated voltages. **(A, B)** Throughout the tested voltage range the increase in T_{inact} remained high and statistically significant for Ca_V3.3-I860N, -I1306T, and -I1425M; while the modest elevation of T_{inact} of I860M did not reach statistical significance. Mean±SEM; *P*-values calculated with repeated measures ANOVA and the Holm-Sidak test for multiple comparisons.

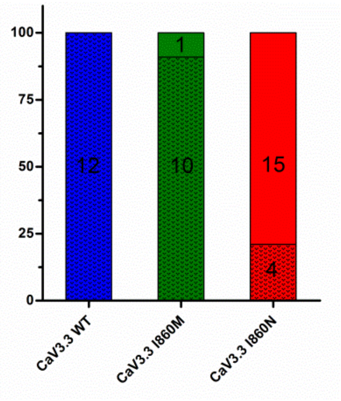

Supplementary Figure S6. AP firing causes cellular stress in chromaffin cells expressing Cav3.3-I860N. Fraction of chromaffin cells with initially good seals that showed increasing leak currents (>50 pA) and dropping membrane resistance (<40 mOhm) during three minutes current clamp recordings. While all WT cells and 10/11 cells expressing Ca_V3.3-I860M had stable membranes, 15/19 chromaffin cells expressing Ca_V3.3-I860N experienced a dramatic loss of seal quality. (WT n=12, I860M n=11, I860N n=19; Chi-Square Test, p< 0.0001).

References

Bouman A, Waisfisz Q, Admiraal J, van de Loo M, van Rijn RR, Micha D, et al. Homozygous DMRT2 variant associates with severe rib malformations in a newborn. Am J Med Genet A 2018; 176: 1216–21.

Cain SM, Snutch TP. Contributions of T-type calcium channel isoforms to neuronal firing. Channels 2010; 4: 475–82.

Calorio C, Gavello D, Guarina L, Salio C, Sassoè-Pognetto M, Riganti C, et al. Impaired chromaffin cell excitability and exocytosis in autistic Timothy syndrome TS2-neo mouse rescued by L-type calcium channel blockers. J Physiol 2019; 597: 1705–33.

Case DA, Ben-Shalom IY, Brozell SR, Cerutti DS, Cheatham TEI, Cruzeiro VWD, et al. AMBER 2018. Univ California, San Fr 2018

Cazade M, Bidaud I, Lory P, Chemin J. Activity-dependent regulation of T-type calcium channels by submembrane calcium ions. Elife 2017; 6: 1–20.

Courel M, Vasquez MS, Hook VY, Mahata SK, Taupenot L. Sorting of the neuroendocrine secretory protein Secretogranin II into the regulated secretory pathway: role of N- and C-terminal alpha-helical domains. J Biol Chem 2008; 283: 11807–22.

Destexhe A, Contreras D, Steriade M, Sejnowski TJ, Huguenard JR. In vivo, in vitro, and computational analysis of dendritic calcium currents in thalamic reticular neurons. J Neurosci 1996; 16: 169–85.

Dickson CJ, Madej BD, Skjevik AA, Betz RM, Teigen K, Gould IR, et al. Lipid14: The Amber Lipid Force Field. J Chem Theory Comput 2014; 10: 865–79.

Gibson KM, Nesbitt A, Cao K, Yu Z, Denenberg E, DeChene E, et al. Novel findings with reassessment of exome data: implications for validation testing and interpretation of genomic data. Genet Med 2018; 20: 329–36.

Gomora JC, Murbartian J, Arias JM, Lee JH, Perez-Reyes E. Cloning and expression of the human {T-type channel Ca(v)3.3}: insights into prepulse facilitation. Biophys J 2002; 83: 229–41.

Grabner M, Dirksen RT, Beam KG. Tagging with green fluorescent protein reveals a distinct subcellular distribution of L-type and non-L-type Ca2+ channels expressed in dysgenic myotubes. Proc Natl Acad Sci U S A 1998; 95: 1903–8.

Harms FL, Parthasarathy P, Zorndt D, Alawi M, Fuchs S, Halliday BJ, et al. Biallelic loss-of-function variants in TBC1D2B cause a neurodevelopmental disorder with seizures and gingival overgrowth. Hum Mutat 2020; 41: 1645–61.

Hines ML, Carnevale NT. The NEURON simulation environment. Neural Comput 1997; 9: 1179–209.

Houweling AC, Beaman GM, Postma A V, Gainous TB, Lichtenbelt KD, Brancati F, et al. Loss-of-function variants in myocardin cause congenital megabladder in humans and mice. J Clin Invest 2019; 129: 5374–80.

Huguenard JR, Prince DA. A novel T-type current underlies prolonged Ca(2+)-dependent burst firing in GABAergic neurons of rat thalamic reticular nucleus. J Neurosci 1992; 12: 3804–17.

Ioannidis NM, Rothstein JH, Pejaver V, Middha S, McDonnell SK, Baheti S, et al. REVEL: An Ensemble Method for Predicting the Pathogenicity of Rare Missense Variants. Am J Hum Genet 2016; 99: 877–85.

Jagadeesh KA, Wenger AM, Berger MJ, Guturu H, Stenson PD, Cooper DN, et al. M-CAP eliminates a majority of variants of uncertain significance in clinical exomes at high sensitivity. Nat Genet 2016; 48: 1581–6.

Jo S, Lim JB, Klauda JB, Im W. CHARMM-GUI Membrane Builder for Mixed Bilayers and Its Application to Yeast Membranes. Biophys J 2009; 97: 50–8.

Kircher M, Witten DM, Jain P, O'Roak BJ, Cooper GM, Shendure J. A general framework for estimating the relative pathogenicity of human genetic variants. Nat Genet 2014; 46: 310–5.

Lee J, Patel DS, Ståhle J, Park S-J, Kern NR, Kim S, et al. CHARMM-GUI *Membrane Builder* for Complex Biological Membrane Simulations with Glycolipids and Lipoglycans. J Chem Theory Comput 2019; 15: 775–86.

Li H, Durbin R. Fast and accurate short read alignment with Burrows-Wheeler transform. Bioinformatics 2009; 25: 1754–60.

Marcantoni A, Vandael DHF, Mahapatra S, Carabelli V, Sinnegger-Brauns MJ, Striessnig J, et al. Loss of Cav1.3 channels reveals the critical role of L-type and BK channel coupling in pacemaking mouse adrenal chromaffin cells. J Neurosci 2010; 30: 491–504.

McKenna A, Hanna M, Banks E, Sivachenko A, Cibulskis K, Kernytsky A, et al. The Genome Analysis Toolkit: a MapReduce framework for analyzing next-generation DNA sequencing data. Genome Res 2010; 20: 1297–303.

Retterer K, Juusola J, Cho MT, Vitazka P, Millan F, Gibellini F, et al. Clinical application of whole-exome sequencing across clinical indications. Genet Med 2016; 18: 696–704.

Rohl CA, Strauss CEM, Misura KMS, Baker D. Protein Structure Prediction Using Rosetta. Methods Enzymol 2004; 383: 66–93.

Schneeberger PE, Nayak SS, Fuchs S, Kutsche K, Girisha KM. Roberts syndrome in an Indian patient with humeroradial synostosis, congenital elbow contractures and a novel homozygous splice variant in ESCO2. Am J Med Genet A 2020; 182: 2793–6.

Wang K, Li M, Hakonarson H. ANNOVAR: functional annotation of genetic variants from high-throughput sequencing data. Nucleic Acids Res 2010; 38: e164.

Wiel L, Baakman C, Gilissen D, Veltman JA, Vriend G, Gilissen C. MetaDome: Pathogenicity analysis of genetic variants through aggregation of homologous human protein domains. Hum Mutat 2019; 40: humu.23798.

Zhao Y, Huang G, Wu Q, Wu K, Li R, Lei J, et al. Cryo-EM structures of apo and antagonist-bound human Cav3.1. Nature 2019: 1–1.
